# Cardiovascular outcomes and safety associated with statin therapy for primary prevention in older adults with type 2 diabetes: A target trial emulation study

**DOI:** 10.1371/journal.pmed.1005136

**Published:** 2026-06-24

**Authors:** Linda Chan, Wanchun Xu, Esther W. Y. Chan, Eric Yuk Fai Wan

**Affiliations:** 1 Department of Family Medicine and Primary Care, School of Clinical Medicine, Li Ka Shing Faculty of Medicine, The University of Hong Kong, Hong Kong SAR, China; 2 Bau Institute of Medical and Health Sciences Education, Li Ka Shing Faculty of Medicine, The University of Hong Kong, Hong Kong SAR, China; 3 Department of Family Medicine, The University of Hong Kong-Shenzhen Hospital, Shenzhen, Guangdong, China; 4 Centre for Safe Medication Practice and Research, Department of Pharmacology and Pharmacy, The University of Hong Kong, Hong Kong SAR, China; 5 Accident and Emergency Department, Queen Mary Hospital, Pok Fu Lam, Hong Kong SAR, China; 6 The University of Hong Kong Shenzhen Institute of Research and Innovation, Shenzhen, China; 7 Centre for Health AI Research and Translation, Li Ka Shing Faculty of Medicine, The University of Hong Kong, Hong Kong SAR, China; 8 Comprehensive Primary Healthcare Collaboratory, Li Ka Shing Faculty of Medicine, The University of Hong Kong, Hong Kong SAR, China; 9 The Institute of Cardiovascular Science and Medicine, Li Ka Shing Faculty of Medicine, The University of Hong Kong, Hong Kong SAR, China; Wuqu’ Kawoq | Maya Health Alliance, GUATEMALA

## Abstract

**Background:**

There is limited evidence on the use of statins for primary prevention of cardiovascular disease (CVD) in older adults with type 2 diabetes due to underrepresentation of this population in randomized controlled trials (RCTs). We aimed to determine the effectiveness and safety of statin therapy for primary CVD prevention among type 2 diabetes patients aged ≥75 years.

**Methods and findings:**

In this cohort study, territory-wide electronic health records (EHRs) from the Hospital Authority Clinical Management System in Hong Kong were used to emulate a sequence of nested target trials. Eligible patients were included in a rolling basis in each calendar month from January 2009 to December 2015, and thus we emulated 84 ‘nested monthly trials’. In each monthly trial, all type 2 diabetes patients aged ≥60 years with elevated low-density lipoprotein cholesterol (≥2.6 mmol/L) in the baseline calendar month were included; patients with a history of type 1 diabetes, CVDs, cancers, muscle-related disorders, or liver dysfunction were excluded from analysis. Eligible individuals were classified into statin initiators or noninitiators based on whether they initiated statin therapy at the time of enrollment. They were categorized into various age groups (60–74, 75–84, ≥85 years) for analysis, with those aged 60–74 years forming a benchmark group to test the validity of the emulated target trial. Patients were followed up until the outcome of interest, death, or the administrative end (December 2018), whichever occurred first. We estimated hazard ratios (HRs) comparing statin use versus nonuse for CVDs, all-cause mortality, muscle-related adverse events (AEs), and liver dysfunction using pooled logistic models, with inverse probability weighting to adjust for time-varying confounders related to treatment adherence, under the assumption of no unmeasured confounding. Propensity score matching was performed on eligible person-trials at baseline, incorporating demographic characteristics, clinical and laboratory parameters, comorbidities, medication history, and healthcare utilization as matching variables. Among 30,804 matched person-trials aged 75–84 years, a significant reduction in the incidence of CVDs (HR 0.69 (95% CI [0.65, 0.75]; *p* < 0.001)) and all-cause mortality (0.65 [0.60, 0.70], *p* < 0.001) was observed. In 3,798 matched person-trials aged ≥85 years, the benefits were consistently observed (CVDs: 0.65 [0.54, 0.77], *p* < 0.001; all-cause mortality: 0.61 [0.52, 0.71], *p* < 0.001). No substantially increased risks for muscle-related AEs or liver dysfunction were observed in both age groups. The effectiveness and safety of statins for the benchmark age group (60–74 years) were also confirmed. The remaining source of bias included the potential misclassification bias due to reliance on diagnosis coding in EHRs, as well as unmeasured confounding relating to lifestyle factors, social determinants, and information on the shared decision-making between physicians and patients.

**Conclusions:**

In type 2 diabetes patients aged ≥75 years, we found that statin use was associated with reduced risks of CVDs and all-cause mortality, including those aged over 85 years. No substantially increased risks for muscle-related adverse events and liver dysfunction were observed. Future studies, including RCTs, are warranted to confirm the effectiveness and safety of statin use in older adults with diabetes, and to determine optimal statin dosing and the comparative efficacy of different statin types, thereby improving CVD prevention in this population.

## Author summary

**Why was this study done?:**

- Statins are first-line therapy for dyslipidemia due to their efficacy for the primary prevention of CVD in type 2 diabetes patients.
- Existing evidence on statin use for primary prevention among type 2 diabetes patients aged ≥75 years is inconclusive due to the under-representation of this population in randomized controlled trials.

**What did the researchers do and find?:**

- We used real-world data from electronic health records (EHRs) with the aim of emulating a target trial to assess the long-term effectiveness and safety of statin therapy for primary CVD prevention in type 2 diabetes patients ≥75 years.
- In patients with type 2 diabetes aged ≥ 75 years, we found statin therapy was associated with substantial reductions in cardiovascular disease incidence and all-cause mortality. This benefit persisted among individuals aged ≥ 85 years, without evidence of substantially increased risk for muscle-related AEs or liver dysfunction.

**What do these findings mean?:**

- Our study reveals potential benefits of statin therapy for primary prevention of cardiovascular disease and reduction of all-cause mortality in individuals with type 2 diabetes aged ≥ 75 years, including those ≥ 85 years, providing valuable evidence for clinicians and patients when considering statin use versus potential side effects.
- These findings align with international guidelines supporting patient‑centered, individualized statin therapy for individuals with type 2 diabetes aged ≥ 75 years, emphasizing shared decision‑making and a shift from age‑based thresholds toward personalized treatment.
- The study was conducted using EHRs, which did not capture information on lifestyle factors, social circumstances, or shared decision-making between physicians and patients. In addition, potential misclassification due to reliance on diagnostic codes cannot be ruled out. These inherent limitations of EHR-based research may have influenced the findings.

## Abstract 3

In a target trial emulation study, Linda Chan and Wanchun Xu investigate the cardiovascular outcomes and safety of statin therapy in older adults with type 2 diabetes.

## Introduction

Cardiovascular disease (CVD) and type 2 diabetes are leading causes of death worldwide [1], with the risk of developing CVD about doubled among type 2 diabetes patients [2]. Alarmingly, 853 million people are predicted to have type 2 diabetes by 2050 [3]. Owing to the aging global population [4], the proportion of type 2 diabetes patients aged ≥75 years is expected to increase, raising concerns about CVD prevention and management among this vulnerable group. Statins are first-line therapy for dyslipidemia due to their efficacy for the primary prevention of CVD in type 2 diabetes patients without manifest disease [5]. However, despite well-established benefits of statin therapy among diabetics as reflected in international guidelines [6–10], there is a lack of robust evidence supporting its effectiveness and safety in those aged ≥75 years [7]. Few type 2 diabetes patients representing this age group have been enrolled in primary prevention trials [7], limiting the generalizability of these studies’ findings among patients of this age. Furthermore, concerns about potential adverse effects of statins among older individuals have been highlighted, such as myopathy and impaired liver function [11,12].

Existing evidence on statin use for primary prevention among type 2 diabetes patients aged ≥75 years is limited to subgroup analysis within a randomized controlled trial (RCT) [13] or observational cohort studies [14–16], with inconsistent findings. Some studies have reported reductions in CVD risk with statin therapy in older type 2 diabetes patients [14–16]. However, no significant benefit was reported in the Prospective Study of Pravastatin in the Elderly at Risk (PROSPER) trial possibly due to an insufficient number of type 2 diabetes patients aged 70–82 being included and a shorter mean follow-up period of 3 years impacting the interpretation of the treatment effect for statins [13]. Furthermore, subgroup analysis in PROSPER excluded patients >82 years [13], limiting the generalizability of the findings to those in this age group. Findings from observational studies were limited by small sample sizes [14], potential biases in selection [14], treatment adherence and time-varying confounders [15] plus challenges in generalizing to ethnically different populations [14].

To address these research gaps, we used real-world data from electronic health records (EHRs) with the aim of emulating a target trial to assess the long-term cardiovascular outcomes and safety associated with statin therapy for primary CVD prevention in type 2 diabetes patients ≥75 years, with a parallel analysis in those aged 60–74 years serving as a positive control to provide benchmark comparisons.

## Methods

### Ethics statement

Ethics approval was obtained from The University of Hong Kong/Hospital Authority Hong Kong West Cluster Institutional Review Board (ref: UW 19-362). As this study used anonymous and de-identified data only, obtaining individual consent from each participant was not required.

### Overview and study participants

**Fig 1:**
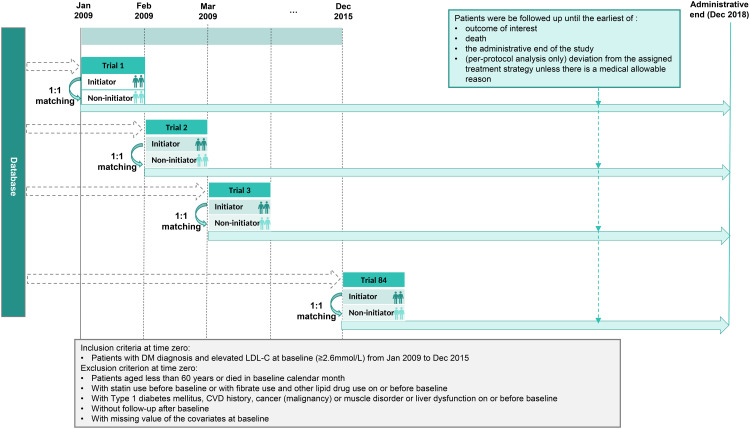
Diagram of the sequential target trial emulation. Eligible patients were included on a rolling basis in each calendar month from January 2009 to December 2015. Participants were classified into the statin initiator or noninitiator groups based on their prescription records at baseline. Propensity score matching was applied to the eligible person-trials at baseline, where the initiators and noninitiators were matched in a 1:1 ratio within each age group (60-74, 75-84, ≥ 85 years).

**Fig 2:**
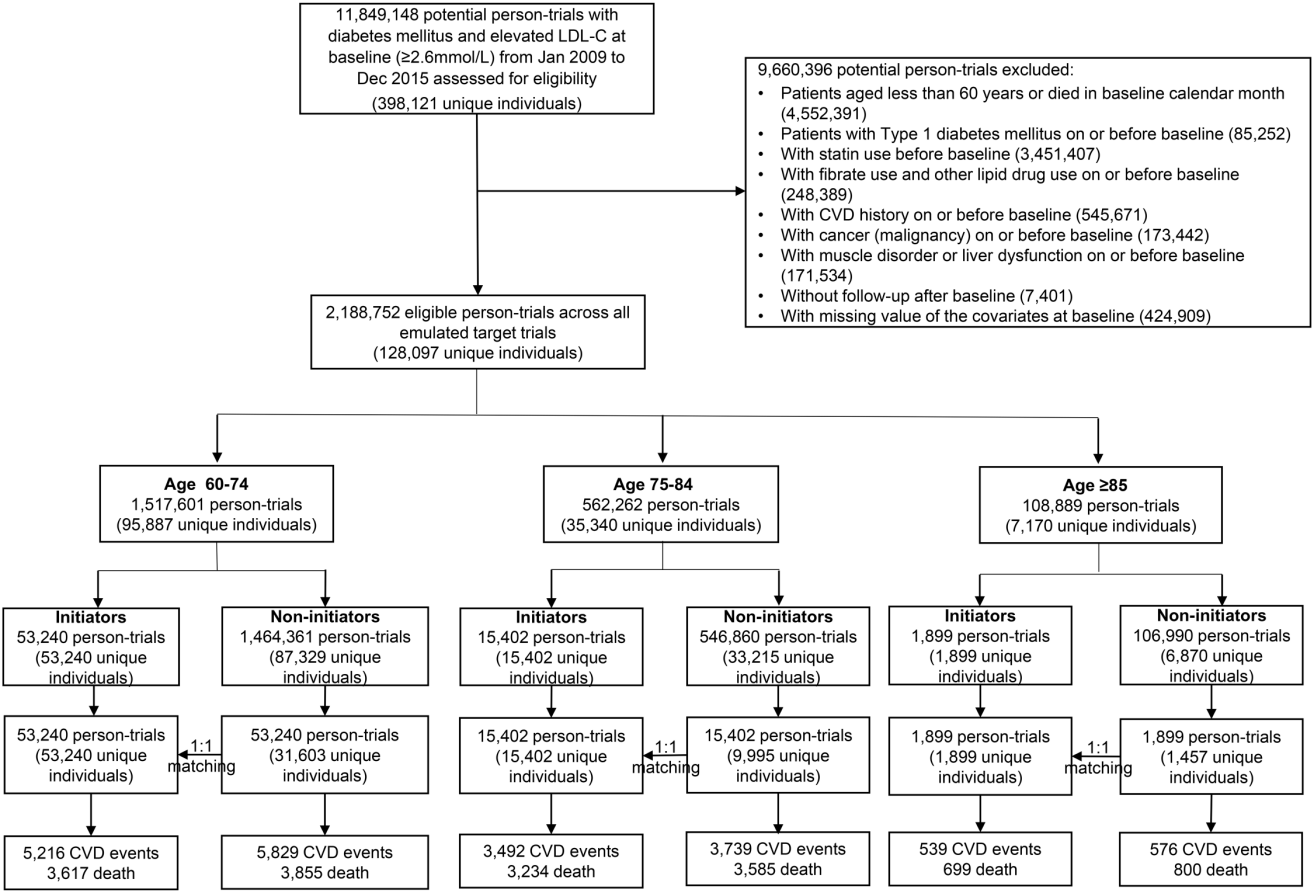
Flowchart of person-trials in the analysis. The unit of analysis in sequentially emulated trials is each individual in each trial (i.e., a person-trial). The number of individuals (in the parentheses) do not sum to the total number of unique individuals because some eligible individuals contributed to different arms in different emulated trials.

To test the hypothesis that statin therapy is effective in the primary prevention against CVD in the old adults with Type 2 diabetes, a sequence of nested target trials (protocol in S1 Appendix) was emulated (Table 1 and Fig 1) using EHRs from the Clinical Management System (CMS) of the Hong Kong (HK) Hospital Authority, with an administrative end of December 2018. The CMS database is an integrated clinical workstation, giving clinicians access to all available electronic clinical information for routine clinical practice. The analysis included all type 2 diabetes patients aged ≥60 years with elevated low-density lipoprotein cholesterol (LDL-C) ≥2.6 mmol/L in each calendar month from January 2009 to December 2015. Criteria for eligibility are shown in more detail in Fig 2. The study protocol is provided in S2 Appendix, which includes the pre-specified data analysis plan and the analyses revised or added after peer review.

**Table 1:** Specification and emulation of target trials.

Protocol component	Target trial specification	Target trial emulation
Eligible criteria	With documented type 2 diabetes diagnosis and LDL-C ≥ 2.6 mmol/L at baseline. Age ≥ 60 years old (categorized into three age groups to conduct separate analysis: 60–74, 75–84, and ≥85 years old). Not using statin before baseline. Not using other lipid regulating drugs on or before baseline. No history of type 1 diabetes, CVDs, cancer, liver dysfunction, or muscle related disorder on or before baseline. The baseline characteristics regarding the covariates for each study participant are recorded in the baseline calendar month. The eligible participants are enrolled on a rolling basis from January 2009 to December 2015.	Same as for the target trials. Patients should have at least one follow-up after baseline. Patients with incomplete information for the baseline covariates were excluded from analysis.
Treatment strategy	Initiate statin use vs. not initiate statin use. Physician will decide whether to stop or start statin therapy when the contraindication (myopathies and liver dysfunction) occurred in the treatment group, or the indication of hyperlipidemia (the most recent LDL-C ≥ 2.6 mmol/L; or LDL-C ≥ 1.8 mmol/L after the first incidence of coronary heart disease, stroke, or heart failure) occurred in the control group during the follow-up period.	Same as for the target trials. 1-month gap was given for the ascertainment of statin discontinuation. Information on diseases and LDL-C levels related to the indication and contraindication for statin use was updated by month in the dataset based on the most recent records.
Treatment assignment	Subjects are randomly assigned to a treatment strategy at baseline and will be aware of the treatment strategy they are assigned to.	Participants were classified into different groups according to the prescription records at baseline. Propensity score matching was applied to the eligible participants, where the initiators and noninitiators were matched in a 1:1 ratio within each age group.
Outcomes	Incidence of cardiovascular diseases (CVDs) as a composite outcome of myocardial infarction, heart failure and stroke, all-cause mortality, and major adverse events. Specific subtypes of CVDs: • Myocardial infarction • Heart failure • Stroke Major adverse events: • Muscle related AE • Liver dysfunction	Same as for the target trial.
Follow-up	From baseline until the occurrence of death, the outcome of interest, or 31 December 2018, whichever comes first.	Same as for the target trial.
Target estimand	Average treatment effects on the treated	Same as for the target trial. Sensitivity analysis was performed on the average treatment effect in the unmatched sample.
Statistical analysis	Intention-to-treat (ITT) analysis. Per-protocol analysis: censored the patients when they deviated from the treatment strategy. Subgroup analysis by sex and Charlson Comorbidity Index (CCI < 8/ ≥ 8) at baseline.	Same as for the target trials, where the ITT analysis and per-protocol analysis were conducted via sequence trial emulation. The statistical model was adjusted by the baseline covariates and the inverse probability weighting was applied to account for the selection bias introduced by the artificial censoring in the per-protocol analysis. Each person-trial was additionally adjusted by a time-varying inverse probability weight of not dying to adjust for the potential bias arising from the competing event of death.

### Target trial emulation

The protocol of the target trial.

#### Eligible criteria.

Eligible individuals include all type 2 diabetes patients aged ≥60 years with elevated LDL-C ≥ 2.6 mmol/L at baseline. An LDL-C cutoff of 2.6 mmol/L is chosen as this represents the optimal treatment target for type 2 diabetes patients with no prior history of CVD according to local guidelines [17]. Patients who use statins before baseline, or fibrates or any other lipid-lowering drugs classified under the British National Formulary code 2.12 (e.g., niacin and its derivatives, ezetimibe, etc.) before baseline are excluded from the analysis. The eligible participants are enrolled on a rolling basis from January 2009 to December 2015. The baseline characteristics regarding the covariates for each study participant are recorded in the baseline calendar month (details in S1 Appendix).

#### Intervention strategies.

The trial compares two specific treatment strategies: (1) Initiating statin therapy and remain on treatment during the follow-up; (2) Not initiating statin therapy during the follow-up period. Patients in treatment group can stop statin therapy if contraindications arise, or start statin therapy in the control group if indications of hyperlipidemia occurred. Statin therapy is defined as the treatment with simvastatin, atorvastatin, fluvastatin, rosuvastatin, lovastatin, pitavastatin, and/or pravastatin.

#### Treatment assignments.

Eligible participants are randomly assigned to one of the two treatment strategies at baseline. Participants will be aware of the treatment strategy to which they are assigned.

#### Follow-up period.

Patients are followed up from the time of treatment assignment until the outcome of interest, death, or 31 December 2018, whichever comes first.

#### Outcome definition.

The primary outcome of interest is the overall incidence of CVDs (a composite outcome of myocardial infarction (MI), heart failure, and stroke). Secondary outcomes include specific CVD subtypes (MI, heart failure, and stroke), all-cause mortality, muscle-related adverse events (AEs), and liver dysfunction. Heart failure is included as a secondary outcome in this study and also included in the primary composite outcome as it is increasingly recognized as an important outcome in the studies of statin therapy, including the ongoing trials of PRagmatic EValuation of evENTs And Benefits of Lipid-lowering in oldEr adults (PREVENTABLE) and STAtins in Reducing Events in the Elderly (STAREE) in older adults [18,11].

#### Causal contrasts of interest.

To compare the two treatment strategies specified above, we estimate the intention-to-treat (ITT) effect and the per-protocol effect. The ITT analysis compared the risk for outcome incidence between the statin initiators and noninitiators defined by their treatment strategy at baseline. The per-protocol analysis compared risks for outcomes of interest among the individuals who had been adhered to the assigned treatment strategies between arms.

#### Analysis plan.

The ITT and per-protocol effects on the prevention of CVDs and all-cause mortality are estimated in the three age groups. An estimate of the ITT HR is obtained by fitting a pooled logistic model for the outcome incidence, including the indicators of the assigned strategy (statin initiation at baseline), follow-up period (linear and quadratic terms) and the aforementioned covariates at baseline. In per-protocol analysis, the person-trials are artificially censored when the participant deviated from their assigned strategy unless the patient developed an indication [17,19] or contraindication [20] for statin therapy (details in Method A in S3 Appendix). Specifically, for statin initiators, they would be allowed to discontinue statin therapy when they experience muscle-related AEs or liver dysfunction. For the statin noninitiators at baseline, they would be allowed to initiate statins in response to an indication of hyperlipidemia. That is, statin initiation is allowed during the follow-up period if the most recent LDL-C measurement is ≥ 2.6 mmol/L in the absence of coronary heart disease, stroke, or heart failure, or ≥ 1.8 mmol/L following the first occurrence of any of the aforementioned CVD events during the follow-up period. To adjust for selection bias resulting from the artificial censoring process described above, each person-trial is weighted at each time point by the inverse probability of receiving their assigned treatment strategy, conditional on the baseline and time-varying covariates. The pooled logistic model is fitted to predict the probability of receiving statin therapy at each time point. To adjust for potential bias arising from the competing events (i.e., death in the present study), each person-trial is additionally adjusted by a time-varying inverse probability weight of not dying. As the treatment strategies in the per-protocol analysis described above are adaptive to the time-varying clinical characteristics (i.e., LDL-C levels and the occurrence of any subtypes of CVDs), we adopt the nonstabilized inverse probability weight, rather than stabilized weight, for estimation of the per-protocol effect after peer review. This change of analytical choice aims to avoid the potential bias introduced by the numerator of stabilized weight. The cumulative product of the estimated weights up to each time point is used as the final weight for each person-trial, which is truncated at 10 to avoid the influence of outliers in the estimated weights when estimating the result [21,22]. Finally, a pooled logistic model is fitted to estimate the hazard ratio (HR) for the outcomes between continuous statin therapy and never using statins during the follow-up period, which includes the indicators of the assigned treatment strategy, month of follow-up (linear and quadratic term), and the baseline covariates, with the adjustment of nonstabilized weight mentioned above. Additional details regarding the per-protocol analysis can be found in Method A in S3 Appendix). For HR estimates in both ITT and per-protocol analysis, standard errors are obtained from the pooled logistic model fitted in the matched study participants to generate the CI. As the observations at different timepoints within the same person-trial are correlated, clustered sandwich estimator is adopted to derive the robust standard errors allowing for clustering [23].

We estimate the absolute risk of outcome incidence by fitting the aforementioned pooled logistic model for the causal effect estimation, incorporating the added product terms between the treatment indicator and time (linear and quadratic terms). The cumulative risk is standardized to the empirical distribution of the confounders at baseline in the entire population. Nonparametric bootstrapping with 500 samples is used to obtain the 95% confidence intervals of the absolute risk difference. For the per-protocol analysis, both the weighting models for obtaining inverse probability weights and the outcome models for absolute risk estimates are included in the bootstrap program. The Number Needed to Treat (NNT) to prevent one additional outcome is calculated based on the estimated 5-year and 10-year risk difference of overall CVD.

### Emulation of the target trial using observational data

#### Eligible criteria.

We applied the eligible criteria to the extracted data from CMS. Our available prescription records for analysis also dated back to January 2006, ensuring that participants included in our study had a minimum 3-year look-back period for washout. Likewise, individuals with a history of type 1 diabetes, CVDs, cancer, as well as muscle-related disorder or liver dysfunction were excluded. Eligible patients were required to have had at least one follow-up visit record after baseline. Patients with incomplete data for the study variables at baseline were also excluded (Data completion rate: Table A in S3 Appendix). A sequence of nested target trials was emulated to obtain a desirable number of statin initiators and cases. Eligible patients were included in a rolling basis in each calendar month from January 2009 to December 2015, and thus we emulated 84 ′nested monthly trials’. The patients were categorized into different age groups (60–74, 75–84, and ≥85 years) for analysis. The analysis of those aged between 60 and 74 years served as a benchmark to test the validity of our emulated trial since the effect of statin therapy was well-established in this population [24,25].

#### Intervention strategies and treatment assignments.

Participants were classified into the statin initiator or noninitiator groups based on their prescription records at baseline. Propensity score matching was applied to the eligible person-trials at baseline, where the initiators and noninitiators were matched in a 1:1 ratio within each age group (60–74, 75–84, ≥85 years). A 1:1 nearest neighbor propensity score matching without replacement was adopted, with a propensity score estimated using logistic regression of the treatment on the covariates. The caliper value was set at 0.2 times the standard deviation of the propensity score. The matching factors included demographic characteristics (sex and age), clinical parameters plus blood profile (systolic blood pressure, diastolic blood pressure, hemoglobin A1c, LDL-C, high-density lipoprotein cholesterol, total cholesterol, and estimated glomerular filtration rate), comorbidities (hypertension, peripheral vascular disease, atrial fibrillation, chronic obstructive pulmonary disease, renal disease, dementia, obesity, and Charlson Comorbidity Index (CCI)), drug history within 1 year before baseline (aspirin, insulin, oral antidiabetic drugs, β-blockers, calcium channel blockers, diuretics, and angiotensin-converting enzyme inhibitors), service utilization within the prior 1 year (Specialist Out-Patient Clinic attendance and hospitalization) and lifestyle behavior (smoking status), which were incorporated with the indicator of calendar month at baseline. The covariates were selected due to their potential role as confounders between exposure and outcomes, as supported by literature [15,26]. Due to the lack of randomization, the assumption of exchangeability conditional on baseline covariates is required for the identification of causal effects. We examined the distribution of propensity scores between treatment groups to evaluate whether the assumptions of exchangeability and positivity were satisfied [27,28].

#### Follow-up period.

The follow-up period will mirror the target trial. Given that medication compliance was not available in the electronic health record, the continuity of statin prescription was assessed for the ascertainment of adherence to the assigned treatment strategies, allowing a 1-month grace period to confirm statin discontinuation.

#### Outcome definition.

Case definition was based on the International Classification of Primary Care, 2nd Edition (ICPC-2) and International Classification of Diseases, 9th Revision, Clinical Modification (ICD-9-CM), or relevant clinical parameters (Table B in S3 Appendix). After peer review, we also analyzed the composite outcome of major CVD diseases without heart failure as a sensitivity analysis for comparison, and expanded the assessment of muscle-related AEs to include myalgia, myositis, myopathy, and rhabdomyolysis. We excluded patients with preexisting diagnosed outcomes of interest on or before baseline to ensure the outcome events were restricted to the new incident cases occurring after baseline. The patients with documented old MI diagnosis (ICD-9 code: 412) or late effects of cerebrovascular disease (ICD-9 code: 438.x) on or before baseline were also excluded. For consistency, these two codes were also included in the case definitions for outcome events for MI and stroke. After peer review, we also conducted a sensitivity analysis where these two codes were excluded from the case definition of outcome events for comparison. Potential bias of undiagnosed disease might exist for the participants who experienced the outcome incidence within the first year of follow-up. After peer review, we retained these patients in the main analysis and conducted a sensitivity analysis by excluding these patients to test the potential bias.

#### Causal contrast(s).

Same as in the target trial.

#### Analysis plan.

The ITT and per-protocol analyses are the same as specified in the target trial. The last observation carried forward method was employed to handle missing values of the time-varying clinical parameters during the follow-up period. The E-value is calculated to assess the robustness of the estimated results to the potential unmeasured confounding [29].

#### Subgroup analyses and sensitivity analyses.

Subgroup analyses for the risk of overall CVD incidence and all-cause mortality were conducted based on sex and CCI (<8/≥8) at baseline. Several sensitivity analyses were conducted. (1) To examine if the length of the grace period for the ascertainment of statin discontinuation would have an impact on the results, a sensitivity analysis was conducted by extending this gap from 1 month to 3 months in the per-protocol analysis. (2) To evaluate the residual confounding by indication, the patients with familial hypercholesterolemia at baseline were excluded in another sensitivity analysis. (3) An additional sensitivity analysis was conducted regarding the weight truncation at the cutoff of 20. After peer review, we additionally performed the following sensitivity analyses to test the robustness of our results: (4) To test whether the choice of LDL-C cutoff might influence the results, we performed a sensitivity analysis on all patients with T2DM. (5) Regarding the competing event of death, we conducted a sensitivity analysis to estimate the total effects, where death was not considered as a censoring event [30]. (6) As mentioned, a sensitivity analysis was conducted by excluding the participants who had the outcome incidence within the first year of follow-up. (7) As the main analysis was conducted in the matched sample, the estimation better reflects Average Treatment Effect on the Treated (ATT) rather than Average Treatment Effect (ATE). To investigate whether the estimated ATT would materially differ from the ATE in our study, we additionally performed a sensitivity analysis by including all eligible study participants before matching. (8) A sensitivity analysis was conducted by removing the exclusion criterion of requiring at least one follow-up visit after baseline. Instead, the person-trials were censored two years after their last recorded visit within the local public healthcare system (i.e., considered them lost to follow-up at this time point) and additionally applied inverse probability weights to account for censoring due to loss to follow-up. (9) For the primary composite outcome, we also analyzed the composite outcome without heart failure for comparison. (10) Finally, for the outcomes of MI and stroke, we performed a sensitivity analysis by excluding the old MI diagnosis (ICD-9 code: 412) or late effects of cerebrovascular disease (ICD-9 code: 438.x) in the case definition for outcome events after baseline.

All analyses were conducted from September to December 2023 using Stata/MP, version 17·0 (StataCorp LLC). Statistical significance was defined as a two-tailed p-value <0·05. This study is reported as per TrAnsparent ReportinG of studies Emulating a Target trial (TARGET) guideline [31] (S1 Checklist).

## Results

Among 11,849,148 person-trials from 84 “trials” (398,121 unique individuals in total) with diabetes diagnosis and elevated LDL-C at baseline from 2009 to 2015, we identified 2,188,752 person-trials eligible for the study, where 70,541 noninitiators were matched to 70,541 initiators in the emulated target trials. This included 106,480 matched person-trials (53,240 pairs) among those aged 60–74 years, 30,804 matched person-trials (15,402 pairs) among those aged 75–84 years, and 3,798 matched person-trials (1,899 pairs) among those aged ≥85 years (Fig 2). Table A in the S3 Appendix presents the percentage of missing data for the baseline covariates before excluding those with incomplete information. The number of eligible person-trials, statin initiators, and the number of CVD events in each trial is shown in S1 Data. The average CCI in each age group mentioned above was around 3.9, 5.9, and 7.3, respectively (Table 2). The baseline characteristics of the eligible person-trials before matching are presented in Table C in S3 Appendix. The distribution of propensity score is presented in Fig A in S3 Appendix.

**Table 2:** Baseline characteristics of eligible person-trials.

	60-74 years			75-84 years			≥ 85 years		
**Initiator**	**Noninitiator**	**SMD**	**Initiator**	**Noninitiator**	**SMD**	**Initiator**	**Noninitiator**	**SMD**
53,240	53,240		15,402	15,402		1,899	1,899	
Age	66.6 (4.2)	66.6 (4.3)	<0.01	79.0 (2.7)	79.0 (2.7)	0.02	87.8 (2.4)	87.7 (2.3)	0.05
Sex (male)	24,209 (45.5%)	24,415 (46%)	<0.01	5,782 (37.5%)	5,780 (38%)	<0.01	558 (29.4%)	569 (30%)	0.01
Smoking	2,394 (4.5%)	2,399 (5%)	<0.01	415 (2.7%)	430 (3%)	<0.01	40 (2.1%)	44 (2%)	0.01
Blood pressure (mmHg)									
SBP	148.2 (16.4)	148.0 (16.3)	<0.01	151.9 (16.5)	151.8 (16.6)	0.01	153.2 (17.2)	153.1 (17.5)	<0.01
DBP	81.8 (9.2)	81.8 (9.3)	<0.01	77.9 (9.4)	77.8 (9.5)	<0.01	76.1 (9.5)	76.7 (10.0)	0.05
HbA1c	7.5 (1.6)	7.5 (1.5)	0.02	7.3 (1.4)	7.3 (1.4)	<0.01	7.0 (1.3)	7.0 (1.3)	<0.01
Lipid profile									
LDL-C									
mmol/L	3.7 (0.7)	3.6 (0.7)	0.04	3.6 (0.7)	3.6 (0.7)	0.05	3.7 (0.7)	3.6 (0.7)	0.06
mg/dL	141.3 (25.6)	140.2 (28.0)	0.04	140.0 (25.7)	138.8 (27.5)	0.05	141.8 (25.7)	140.1 (27.7)	0.06
HDL-C									
mmol/L	1.3 (0.3)	1.3 (0.3)	<0.01	1.4 (0.3)	1.4 (0.4)	0.01	1.4 (0.4)	1.4 (0.4)	0.03
mg/dL	51.6 (12.9)	51.5 (13.3)	<0.01	52.5 (13.5)	52.7 (14.0)	0.01	53.3 (14.2)	53.6 (14.8)	0.03
Total cholesterol									
mmol/L	5.7 (0.8)	5.6 (0.9)	0.03	5.6 (0.8)	5.6 (0.8)	0.03	5.7 (0.8)	5.7 (0.9)	0.04
mg/dL	219.2 (30.9)	218.2 (33.0)	0.03	218.3 (30.6)	217.2 (32.5)	0.03	221.0 (30.8)	219.6 (33.2)	0.04
eGFR	98.6 (24.3)	98.6 (24.6)	<0.01	82.6 (25.1)	82.9 (25.8)	0.01	73.8 (23.1)	73.8 (23.8)	<0.01
Comorbidities									
Charlson Comorbidity Index	3.9 (1.6)	3.9 (1.6)	<0.01	5.9 (2.1)	5.9 (2.1)	0.01	7.3 (2.1)	7.2 (2.1)	0.04
Hypertension	41,527 (78.0%)	41,868 (78.6%)	0.02	13,884 (90.1%)	13,887 (90.2%)	<0.01	1,751 (92.2%)	1,760 (92.7%)	0.02
Obesity	8,911 (16.7%)	9,133 (17.2%)	0.01	1,911 (12.4%)	1,925 (12.5%)	<0.01	161 (8.5%)	173 (9.1%)	0.02
Peripheral vascular disease	156 (0.3%)	138 (0.3%)	<0.01	81 (0.5%)	71 (0.5%)	<0.01	17 (0.9%)	15 (0.8%)	0.01
Atrial fibrillation									
COPD	980 (1.8%)	935 (1.8%)	<0.01	575 (3.7%)	584 (3.8%)	<0.01	100 (5.3%)	107 (5.6%)	0.02
Renal disease	8,364 (15.7%)	8,262 (15.5%)	<0.01	5,769 (37.5%)	5,691 (36.9%)	0.01	999 (52.6%)	969 (51.0%)	0.03
Dementia	121 (0.2%)	103 (0.2%)	<0.01	264 (1.7%)	243 (1.6%)	0.01	80 (4.2%)	89 (4.7%)	0.02
Drug use									
Long-term aspirin users	2,883 (5.4%)	2,956 (5.6%)	<0.01	1,375 (8.9%)	1,365 (8.9%)	<0.01	220 (11.6%)	227 (12.0%)	0.01
Insulin	2,495 (4.7%)	2,458 (4.6%)	<0.01	722 (4.7%)	730 (4.7%)	<0.01	84 (4.4%)	83 (4.4%)	<0.01
Oral antidiabetic drugs	44,429 (83.5%)	44,314 (83.2%)	<0.01	12,639 (82.1%)	12,647 (82.1%)	<0.01	1,491 (78.5%)	1,494 (78.7%)	<0.01
ACEI/ARB	25,292 (47.5%)	25,821 (48.5%)	0.02	8,570 (55.6%)	8,701 (56.5%)	0.02	1,042 (54.9%)	1,032 (54.3%)	0.01
β-blocker	15,423 (29.0%)	15,618 (29.3%)	<0.01	5,326 (34.6%)	5,370 (34.9%)	<0.01	643 (33.9%)	652 (34.3%)	<0.01
Calcium channel blockers	31,432 (59.0%)	31,783 (59.7%)	0.01	11,494 (74.6%)	11,650 (75.6%)	0.02	1,516 (79.8%)	1,540 (81.1%)	0.03
Diuretic	7,003 (13.2%)	6,967 (13.1%)	<0.01	2,798 (18.2%)	2,690 (17.5%)	0.02	359 (18.9%)	343 (18.1%)	0.02
Service utilization									
SOPC attendance in the past 1 year	26,917 (50.6%)	26,830 (50.4%)	<0.01	9,389 (61.0%)	9,276 (60.2%)	0.02	1,163 (61.2%)	1,157 (60.9%)	<0.01
Hospitalization in the past 1 year	5,555 (10.4%)	5,360 (10.1%)	0.01	2,653 (17.2%)	2,609 (16.9%)	<0.01	457 (24.1%)	463 (24.4%)	<0.01

Abbreviations: SBP, systolic blood pressure; DBP, diastolic blood pressure; HbA1c, glycated hemoglobin; LDL-C, low-density lipoprotein cholesterol; HDL-C, high-density lipoprotein cholesterol; eGFR, estimated glomerular filtration rate; COPD, chronic obstructive pulmonary disease; ACEI/ARB, angiotensin-converting enzyme inhibitor and angiotensin II receptor blocker; SOPC, specialist out-patient clinic.

**Fig 3:**
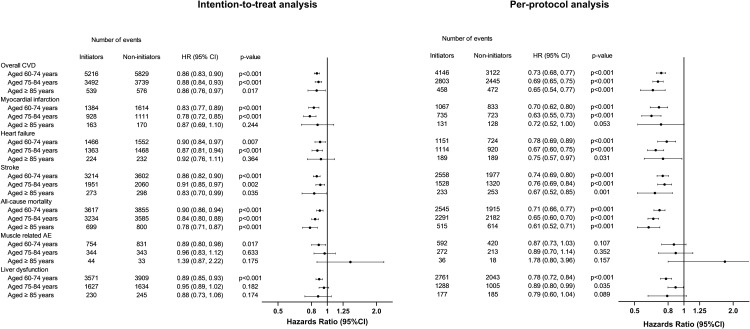
Estimated hazard ratios (95% CI) for cardiovascular diseases and all-cause mortality between statin initiators and noninitiators in three age groups (aged 60–74, 75–84, and ≥85 years old). Analyses adjusted for sex, age, smoking status, hemoglobin A1c, low-density lipoprotein cholesterol, high-density lipoprotein cholesterol, total cholesterol, estimated glomerular filtration rate, comorbidities (including Charlson Comorbidity Index, hypertension, peripheral vascular disease, chronic obstructive pulmonary disease, atrial fibrillation, renal disease, dementia, obesity), drug use (aspirin, insulin, oral antidiabetic drugs, angiotensin-converting enzyme (ACE) inhibitors, β-blockers, calcium channel blockers, diuretics), Specialist Out-Patient Clinic attendance (within 1 year before baseline) and hospitalization (within 1 year before baseline), baseline calendar month, month of follow-up and its square term. *P*-value was derived from the Wald test for the coefficient of the treatment indicator in each pooled logistic regression model. muscle-related AE, muscle-related adverse events.

Table D in S3 Appendix presents the follow-up time and crude incidence rate of each outcome for each treatment strategy in both ITT and per-protocol analysis. During an average follow-up period of 5·5 years (the end of follow-up: the earliest occurrence of overall CVD, death and administrative end), the crude incidence rates of overall CVD incidence for the stain initiators and noninitiators were 17.05 versus 19.49 in those aged 60–74 years, 44.60 versus 49.25 in those aged 75–84 years, and 67.14 versus 75.78 per 1,000 person-years in those aged ≥85 years. The estimated HRs for CVDs, all-cause mortality, and CVD subtypes are presented in Fig 3. In the ITT analysis, the estimated HR of overall CVD incidence for statin initiation was 0.86 (95% CI [0.83, 0.90]; *p* < 0.001) in type 2 diabetes patients aged 60–74 years, compared to 0.88 ([0.84, 0.93]; *p* < 0.001) in those 75–84 years old and 0.86 ([0.76, 0.97]; *p* < 0.017) in those ≥85 years old. The E-values for these estimates were around 1.60 (Table E in S3 Appendix), indicating that to completely explain away the observed association between statin use and the outcome, an unmeasured confounder would need to be associated both with statin use and with the outcome by adjusted risk ratios of at least 1.60 each. In the per-protocol analysis, the estimated HRs for overall CVD incidence were 0.73 ([0.68, 0.77]; *p* < 0.001) in the elderly aged 60–74 years, 0.69 ([0.65, 0.75]; *p* < 0.001) in those 75–84 years old and 0.65 ([0.54, 0.77]; *p* < 0.001) in those ≥85 years old, respectively, and the HRs for all-cause mortality were 0.71 ([0.66, 0.77]; *p* < 0.001), 0.65 ([0.60, 0.70]; *p* < 0.001), and 0.61 ([0.52, 0.71]; *p* < 0.001) accordingly. The observable risk reduction was found for most CVD subtypes in the per-protocol analysis. The coefficients of the weighting model for the treatment history were presented in Table F in S3 Appendix, and the weight distribution by treatment strategies was demonstrated in Fig B in S3 Appendix.

**Fig 4:**
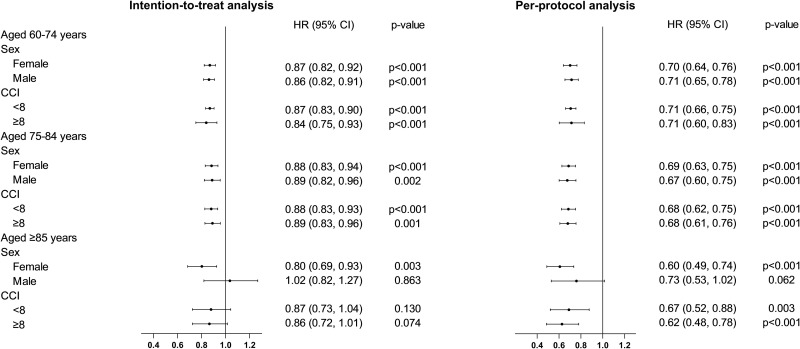
Standardized cumulative incidence curves for cardiovascular diseases over follow-up period between statin users and nonusers (left: intention-to-treat analysis; right: per-protocol analysis). Analyses adjusted for sex, age, smoking status, hemoglobin A1c, low-density lipoprotein cholesterol, high-density lipoprotein cholesterol, total cholesterol, estimated glomerular filtration rate, comorbidities (including Charlson Comorbidity Index, hypertension, peripheral vascular disease, chronic obstructive pulmonary disease, atrial fibrillation, renal disease, dementia, obesity), drug use (aspirin, insulin, oral antidiabetic drugs, angiotensin-converting enzyme (ACE) inhibitors, β-blockers, calcium channel blockers, diuretics), Specialist Out-Patient Clinic attendance (within 1 year before baseline) and hospitalization (within 1 year before baseline), baseline calendar month, month of follow-up and its square term, with the added product terms between treatment and time (linear and quadratic term). Musle-related AE, muscle-related adverse events.

No substantially increased risks for muscle-related AEs and liver dysfunction were consistently observed across all three age groups. Figure 4 shows the standardized cumulative incidence curves for overall CVD incidence for the three age groups. When compared to the never-users, the standardized cumulative incidences of overall CVDs among continuous statin users were lower in all three age groups during the 10-year period (Table G in S3 Appendix). Accordingly, the NNT (95% CI) to prevent one CVD event in 5 years was 15 (12, 20) in those aged 75–84 years and 9 (6, 18) among those aged over 85 years in the per-protocol analysis. For the estimated 10-year absolute risk difference, the NNT in 10 years was 12 to prevent one CVD event in the elderly aged 75–84 years, and 14 in those aged >85 years.

**Fig 5:**
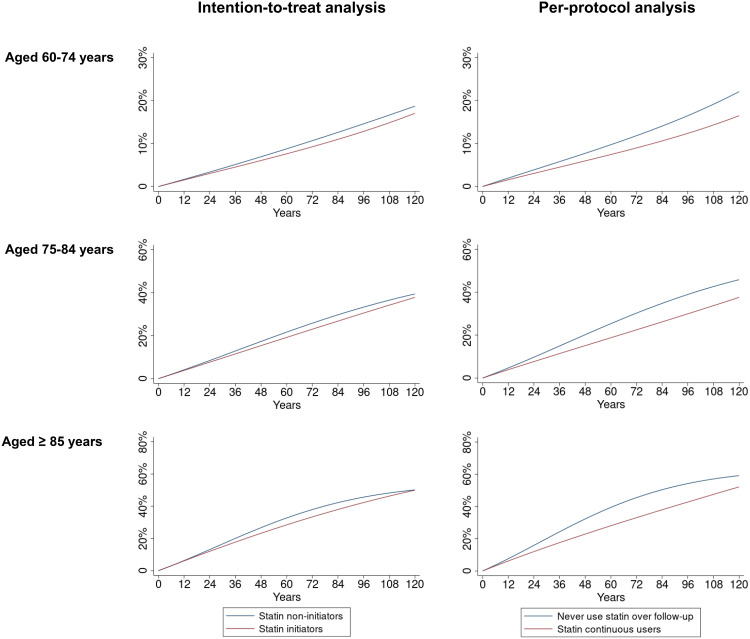
Estimated hazard ratios for overall incidence of cardiovascular diseases between statin initiators and noninitiators in subgroup analysis and sensitivity analysis. Analyses adjusted for sex, age, smoking status, hemoglobin A1c, low-density lipoprotein cholesterol, high-density lipoprotein cholesterol, total cholesterol, estimated glomerular filtration rate, comorbidities (including Charlson Comorbidity Index, hypertension, peripheral vascular disease, chronic obstructive pulmonary disease, atrial fibrillation, renal disease, and dementia, obesity), drug use (aspirin, insulin, oral antidiabetic drugs, angiotensin-converting enzyme (ACE) inhibitors, β-blockers, calcium channel blockers, diuretics), month of follow-up and its square term, Specialist Out-Patient Clinic attendance (within 1 year before baseline) and hospitalization (within 1 year before baseline). *P*-value was derived from the Wald test for the coefficient of the interaction term between the treatment indicator and the subgroup stratifier in each pooled logistic regression model.

The HR estimates in subgroup and sensitivity analyses are presented in Fig 5 and Tables H - R in S3 Appendix. CVD risk reduction was consistently found in the sensitivity analyses. In general, similar estimates for CVD risk reduction were observed according to sex and CCI (<8/≥8) among those aged 60–74 years and 75–84 years, respectively. For those aged ≥85 years, CVD risk reduction by sex and CCI was not significant in the ITT analysis possibly due to the small numbers of patients involved.

## Discussion

In this study, employing real-world population-based data, we assessed the long-term effectiveness and safety of statin use among type 2 diabetes patients ≥75 years old for primary prevention of CVD. Over an average follow-up period of 5.5 years, statin therapy was associated with a reduced risk for CVD and all-cause mortality, while no associated increased risk for major AEs was noted. These findings persisted in type 2 diabetes patients ≥85 years old.

We used patients aged 60–74 years as a positive control in our emulated trial, for whom statin initiation was associated with a decreased risk of overall CVD (HR 0.73 [0.68, 0.79]) in per-protocol analysis. This was consistent with estimates from a meta-analysis [5], where statin use among 18,686 mostly type 2 diabetes patients was associated with a 21% reduction in the incidence of major vascular events (HR 0.79 [0.72, 0·86]) per mmol/L decrease in LDL-C, with similar relative risk reduction in those ≥65 years old versus <65 years. Moreover, a post hoc analysis of the Collaborative Atorvastatin Diabetes Study (CARDS) found a 38% risk reduction (95% CI [−58%, −8%]) in the incidence of first major vascular events in type 2 diabetes patients aged 65–75 years using atorvastatin [32]. A meta-analysis for the U.S. Preventive Service Task Force in 2022 found that statins yielded significant risk reduction in myocardial infarction (HR 0.67, 95%CI [0.60, 0.75]), stroke (0.78, [0.68, 0.90]) and all-cause mortality (HR 0.92 [0.87, 0.98]) in adults without prior cardiovascular events (mean ages ranging from 52–66 years) [33,34]. Based on the real-world data, we found a similar risk reduction in these endpoints in those aged 60–74 years. The consistency of the findings supports the validity of our analytical approach and quality of the data.

Our findings of decreased risks in CVD incidence and all-cause mortality associated with statin initiation in type 2 diabetes patients aged ≥75 years, and particularly those aged ≥85 years, offer additional evidence of the effectiveness of statin therapy in these age groups who are often underrepresented in RCTs [13,32] and meta-analyses [5,35]. In the PROSPER trial [13], no statistically significant effect of pravastatin use or nonuse (HR 1·27 [95% CI 0·9, 1·80]) was noted potentially due to the small numbers of diabetics involved (623 of 5,181 participants). Our results also extend findings from prior observational studies [14,15, 16] with Orkaby and colleagues [16] noting similar risk reductions in CVD (HR 0·76 [95% CI 0·72, 0·80]) and all-cause mortality (HR 0·74 [95% CI 0·72, 0·76]) among a diabetic subset of U.S. veterans aged >75 years. However, this retrospective cohort study focused on the effect of statins for primary prevention of CVD and mortality among U.S. veterans >75 years old more broadly, and although a subset of those had type 2 diabetes, published risk reductions were not age-stratified and no data were reported for those aged <75 years. Similarly, a retrospective cohort study of Spanish primary care patients reported decreased risks of CVD incidence (HR 0·76 [95% CI 0·65, 0·89]) and all-cause mortality (HR 0·84 [95% CI 0·75, 0·94]) with statin therapy among type 2 diabetes patients aged 75–84 years [15]. Conversely, no statistically significant associations were identified for those ≥85 years, possibly due to a small sample size and a relatively low baseline level of LDL-C in this age group [15]. Consistent with Neil and colleagues [32], who found statin therapy over 4 years produced a larger reduction in the absolute risk of cardiovascular events (3·9% versus 2·7%) and a lower NNT to prevent one CVD event (21 versus 33). In older type 2 diabetes patients, we found that the NNT to prevent one CVD event over 5 years was 9 for those ≥85 years old, compared to 14 for 75–84 year olds, and 40 for those aged 60–74 years. The magnitudes of risk reduction for all-cause mortality were greater in the participants aged over 75 years compared to those in the benchmark age group, likely attributable to higher baseline risk within this population [36].

The hypolipidemic, anti-inflammatory, and anti-thrombotic effects of statins [37–40] may be involved in the pharmacological mechanisms supporting our findings. Statins inhibit HMG-CoA reductase, reducing LDL-C levels, thereby exerting anti-atherosclerotic effects [38]. Their anti-inflammatory properties help decrease vascular inflammation and improve endothelial function [37,40], while their anti-thrombotic effects inhibit platelet aggregation and reduce tissue factor expression [39]. These mechanisms could contribute synergistically to the decreased risk of CVD and all-cause mortality observed across all age groups with type 2 diabetes, but further research into underlying biological processes is required.

CVD risk reduction was consistently observed across patient subgroups by sex and comorbidity status in those aged ≥75 years, indicating broad benefits of statin use among this group. Also, no substantially increased risks of myopathies or liver dysfunction were observed across all age groups with type 2 diabetes, endorsing the safety and tolerability of statin therapy in older populations. Both clinical [13] and previous observational [15] studies found no increased risk in adverse effects attributable to statin therapy in their elderly type 2 diabetes participants. A seemingly negative association between statin use and liver dysfunction was observed in some age groups. Although patients with a documented history of liver dysfunction prior to baseline were excluded from the analysis, we acknowledge that potential biases such as indication bias or healthy user bias may still be present, as the information on the shared decision-making between physicians and patients is not captured in the electronic health records for analysis. Therefore, this finding should be interpreted with caution and warrants further research for confirmation.

International guidelines in recent years shift toward highly individualized, shared decision-making on initiating statin therapy for primary prevention in patients with type 2 diabetes aged over 75 years, largely due to a lack of robust clinical trial evidence, including the guideline issued by American Diabetes Association (ADA) [41], and American College of Cardiology/ American Heart Association [9]. For example, the 2026 ADA Standards of Care in Diabetes state that it “may be reasonable” to initiate moderate-intensity statin therapy in those aged >75 years not currently taking a statin. However, this is graded as Level C evidence, reflecting the limited clinical trial data, and requires a careful discussion of potential benefits and risks regarding the patient’s specific health status and life expectancy [41].

Our study highlights the benefits of statin therapy in the primary prevention of CVD and reduction of all-cause mortality among type 2 diabetic individuals aged ≥75 years, and in particular those ≥85 years old, providing evidence for clinicians and patients to consider when weighing statin use against the risk of side effects. Clinical relevance was supported by strongly decreasing NNTs with increasing age to prevent one CVD event in 5 years and in 10 years. Robustness was verified by sensitivity analyses, making our results generalizable to real-world clinical practice, and supporting the notion that age should not be the sole determinant for statin initiation or termination [16]. While polypharmacy remains an important issue, fueling a modern trend towards deprescribing nonessential medications for elderly patients, our study demonstrates the effectiveness and safety of statin therapy for older adults with diabetes, promoting further discussion as to whether statins should be ringfenced against deprescription for some elderly diabetics. These findings are in harmony with international guidelines that patient-centered, bespoke statin therapy can be considered for those with type 2 diabetes aged ≥75 years following physician–patient discussions and support a shift in practice to prioritize individualized treatment decisions over age-based thresholds.

Ongoing trials are aiming to determine the role of statin therapy in preventing cardiovascular events or CVD-related mortality amongst healthy older adults. Two of these, PREVENTABLE in the U.S. and STAREE in Australia are currently investigating statin use for individuals ≥75 years old and ≥70 years old, respectively [11,42]. Despite differences in target population and study design, the similarities in the research questions have the potential to enhance our overall understanding across subgroups of older patients [11], both those with type 2 diabetes and those without. By conducting an emulated target trial, our study leverages the largest patient sample amongst ongoing trials and contributes to our understanding of statin therapy in the prevention of CVD and all-cause mortality amongst a subgroup of older patients with type 2 diabetes. Therefore, at this time while the results of PREVENTABLE and STAREE targeting initially healthy elderly are still pending, our study provides important real-world findings supporting preventive statin therapy among elderly diabetics based on a considerably larger sample size and longer follow-up period compared to those estimated for both RCTs.

Future avenues of research also include examining optimal statin dosing and therapy duration, comparing the efficacy of different statin types, and evaluating the role of combination therapies in older type 2 diabetes patients. Addressing these research gaps could help further refine local plus potentially global clinical practice guidelines and improve the primary prevention of CVD among this rapidly expanding but vulnerable population.

By analyzing the population-based data spanning a period of 10 years, we were able to assess the long-term effectiveness and safety of statin therapy among the diabetic population aged over 75, who have generally been underrepresented in previous clinical trials. Another key strength of this study is the analytical approach we adopted to evaluate the use of sustained statin treatment over the long-term, which accounted for potential confounding factors that may impact medication adherence after the study baseline.

Our study has several limitations to be considered. First, the identification of outcome events depends on the diagnosis coding of ICPC-2 and ICD-9-CM in the EHRs, which may cause misclassification bias. However, it is worth noting that the CMS database used for data extraction has undergone validation in prior studies, where a high coding accuracy was reported in the diagnosis of myocardial infarction and stroke with positive predictive values of 85·4% (95% CI 78·8%, 90·6%) and 91·1% (95% CI 83·2%, 96·1%), respectively [43]. Additionally, as the procedure codes were not included in the initial research protocol for data extraction, the endpoints of percutaneous coronary intervention and coronary artery bypass grafting could not be included in the analysis, which warrants further investigation. Second, we acknowledge that propensity score adjustment does not address all confounding factors. Our study may have been influenced by several unmeasured confounders, including lifestyle factors such as diet and physical activity, social determinants, and the information on the shared decision-making between physicians and patients. For example, patients with low income may be less likely to receive or adhere to treatment [44], yet more likely to have a higher risk of CVDs [45]. Thus, the lack of adjustment for this unmeasured confounding can lead to an overestimation of the beneficial effect of statins. Nevertheless, our analysis in the benchmark age group produced findings consistent with randomized trials conducted on younger patients, indicating that these confounders might have limited effects, as indicated by population-based data. One meta-analysis reported a 50% increased CVD risk for low socio-economic status (SES) compared to high SES (relative risk: 1.49 [95%CI: 1.26, 1.78]) [46]. Therefore, according to the E-value in the quantitative bias analysis, it seems this unmeasured confounding is unlikely to materially alter our conclusions. Third, we did not classify statin use into high-, moderate-, or low-intensity due to data availability. Previous studies have indicated that the prevalence of high-intensity statin prescription in HK was notably lower compared to low- and moderate-intensity statin therapy [47]. It was reported that only 6·2% of patients admitted with acute coronary syndrome in HK were prescribed high-intensity statin therapy [48]. Fourth, we used a nonuser comparator to mirror real‑world decision‑making about statin initiation, but this choice may be subject to other potential indication bias. In our target trial emulation, we aligned time zero with the time point where the eligibility for statin treatment (i.e., the indication of LDL-C ≥ 2.6 mmol/L) is met and treatment strategy is assigned, thereby partially addressing this issue. The results of the sensitivity analysis also suggest that the indication bias related to the history of familial hypercholesterolemia is minimal. Fifth, it is also important to acknowledge that our per-protocol analysis considers continuity of statin prescription rather than actual medication compliance; however, prior research indicates generally good compliance with lipid-lowering agents among the local patients [49]. Finally, the generalizability of our study results to populations with diverse ethnicities may be limited, warranting further research in different settings.

In our target trial emulation of type 2 diabetes patients ≥75 years, statin therapy was found to be associated with reduced risk of CVD and all-cause mortality, including in those aged ≥85 years. Additionally, we did not observe substantially increased risks of muscle‑related AEs or liver dysfunction. Given the observational nature of the study, further research, particularly RCTs, is needed to confirm these findings and to determine optimal statin dosing and comparative efficacy among statin types in this highly heterogeneous population.

## Supporting information

S1 Appendix Protocol for target trial. (DOCX)

S2 Appendix Study protocol. (DOCX)

S3 Appendix Supplementary information. **Table A** - Percentage of missing data for baseline covariates before excluding patients with incomplete information. **Table B** - Case definitions. **Method A** - Details on the per-protocol analysis and the estimation of standardized risk differences. **Table C** - Baseline characteristics of eligible person-trials before matching. **Table D** - Crude incidence rates of the outcome events (S4a Unadjusted crude incidence rate of the outcome events in the person-trials before matching; S4b Crude incidence rates of the outcome events in the final analytical samples). **Table E** - E-value of the outcome estimates in the intention-to-treat analysis. **Table F** - Coefficients of weighting model for treatment history (S6a treatment group; S6b control group). **Table G** - Estimated standardized 5-year and 10-year absolute risk differences for all outcomes. **Table H** - Estimated hazard ratios (95% CI) for all-cause mortality, stratified by sex and Charlson Comorbidity Index (CCI). **Table I** - Sensitivity analysis for using a gap of 3 months for the ascertainment of statin discontinuation in per-protocol analysis: estimated hazard ratio for outcomes of interest. **Table J** - Sensitivity analysis of truncating the inverse probability weights at 20: estimated hazard ratio for outcomes of interest. **Table K** - Sensitivity analysis adjusting for excluding the patients with familial hypercholesterolemia: estimated hazard ratio for outcomes of interest. **Table L** - Sensitivity analysis of including all patients with T2DM (regardless of LDL-C levels): estimated hazard ratio for outcomes of interest. **Table M** - Sensitivity analysis of estimating the total effect for CVD outcomes and adverse events: estimated hazard ratio for outcomes of interest. **Table N** - Sensitivity analysis of excluding the participants who had the outcome within the first year of follow-up: estimated hazard ratio for outcomes of interest. **Table O** - Sensitivity analysis of including all eligible study participants before matching: estimated hazard ratio for outcomes of interest. **Table P** - Sensitivity analysis of censoring patients two years after their last recorded visit within the local public healthcare system: estimated hazard ratio for outcomes of interest. **Table Q** - Sensitivity analysis of the primary composite outcome of major cardiovascular diseases without heart failure. **Table R** - Sensitivity analysis of excluding the diagnosis code of nonincident events in case definition of outcome events. **Fig A** - Distribution of the propensity score. **Fig B** – Distribution of weight distribution by treatment strategies.
(DOCX)

S1 Checklist TARGET checklist. (DOCX)

S1 Data Number of participants, statin initiators, and CVD cases in each target trial in the sequence. (XLSX)

## Abbreviations

ADA: American Diabetes Association
ACE: angiotensin-converting enzyme
AEs: adverse events
ATE: average treatment effect
CCI: Charlson Comorbidity Index
CMS: clinical management system
EHRs: electronic health records
HRs: hazard ratios
ITT: intention-to-treat
MI: myocardial infarction
NNT: number needed to treat
RCTs: randomized controlled trials
SES: socio-economic status
TARGET: TrAnsparent ReportinG of studies Emulating a Target trial.
