# Supplementary material for: Cardiovascular outcomes and safety associated with statin therapy for primary prevention in older adults with type 2 diabetes: A target trial emulation study
Source: PLoS Med. 2026 Jun 24;23(6):e1005136. doi: 10.1371/journal.pmed.1005136 (PMC13293410; doi:10.1371/journal.pmed.1005136)
Supplement: S1 Appendix — (DOCX) [file pmed.1005136.s001.docx]

**Appendix I. Protocol for the target trial**

| Title | Cardiovascular outcomes and safety associated with statin therapy for primary prevention in older adults with type 2 diabetes |
| --- | --- |
| Trial registration | Not applicable |
| Author details | Linda Chan, MD,  Wanchun Xu, PhD,  Esther W. Chan, PhD,  Eric Yuk Fai Wan, PhD |

**1. Background**

Type 2 diabetes (T2D) is a prevalent condition around the world. T2D patients are typically at an elevated risk of developing cardiovascular disease (CVD). As one of the most commonly prescribed lipid-lowering agents, statins are extensively used to mitigate the risk of CVDs in T2D patients with hyperlipidemia. However, there is a lack of consensus regarding the use of statins for primary prevention in T2D patients with advanced age, as limited robust evidence exists on statin use for CVD primary prevention among the older (75-84 years) and very old (≥85 years) T2D patients.

**2. Objective**

To evaluate the effectiveness and safety of statin use for primary prevention against CVD and all-cause mortality in the old (75-84 years) and very-old (≥85 years) T2D patients with hyperlipidemia.

**3. Eligibility Criteria**

Eligible individuals include all type 2 diabetes patients aged ≥60 years with elevated LDL-C ≥2.6 mmol/L at baseline. Patients who had used statins before baseline, or fibrates or any other lipid-lowering drugs classified under the British National Formulary code 2.12 (e.g., niacin and its derivatives, ezetimibe, etc.) before baseline are excluded from the analysis. The eligible participants are enrolled on a rolling basis from January 2009 to December 2015. The baseline characteristics regarding the covariates for each study participant are recorded in the baseline calendar month, which include demographic characteristics (sex and age), clinical parameters plus blood profile (systolic blood pressure, diastolic blood pressure, hemoglobin A1c, LDL-C, high-density lipoprotein cholesterol, total cholesterol, and estimated glomerular filtration rate), comorbidities (hypertension, peripheral vascular disease, atrial fibrillation, chronic obstructive pulmonary disease, renal disease, dementia, obesity, and Charlson Comorbidity Index), concomitant medication use (aspirin, insulin, oral antidiabetic drugs, β-blockers, calcium channel blockers, diuretics, and angiotensin-converting enzyme inhibitors), service utilization in the prior 1 year before randomization (Specialist Out-Patient Clinic attendance and hospitalization) and lifestyle behaviour (smoking status).

**4. Treatment strategies**

The trial compares two specific treatment strategies:

(1) Initiating statin therapy and remaining on treatment during the follow-up;

(2) Not initiating statin therapy during the follow-up period.

Patients in the treatment group can stop statin therapy if contraindications arise, or start statin therapy in the control group if indications of hyperlipidaemia occur. Specifically, for statin initiators, they would be allowed to discontinue statin therapy when they experienced muscle-related adverse events or liver dysfunction. For the statin non-initiators at baseline, they would be allowed to initiate statins in response to an indication of hyperlipidemia. That is, statin initiation was allowed during the follow-up period if the most recent LDL-C measurement was ≥ 2.6 mmol/L in the absence of coronary heart disease, stroke, or heart failure, or ≥ 1.8 mmol/L following the first occurrence of any of the aforementioned CVD events during the follow-up period.

Statin therapy was defined as the treatment with simvastatin, atorvastatin, fluvastatin, rosuvastatin, lovastatin, pitavastatin, and/or pravastatin.

**5. Treatment assignment**

Eligible participants are randomly assigned to one of the two treatment strategies at baseline. Participants will be aware of the treatment strategy to which they are assigned.

**6. Follow-up period**

Patients are followed up from the time of treatment assignment until the outcome of interest, death, or 31 December 2018, whichever comes first.

**7. Outcome definition**

Primary outcome

- The overall incidence of cardiovascular diseases (CVDs), defined as a composite of myocardial infarction (MI), heart failure, and stroke.

Secondary outcomes

- Individual CVD subtypes (MI, heart failure, and stroke)
- All-cause mortality
- Muscle-related adverse events
- Liver dysfunction

**8. Causal contrasts of interest**

To compare the two treatment strategies specified above, we estimate the intention-to-treat (ITT) effect and the per-protocol effect. The ITT analysis compared the risk for outcome incidence between the statin initiators and non-initiators defined by their treatment strategy at baseline. The per-protocol analysis compared risks for outcomes of interest among the individuals who adhere to the assigned treatment strategies between arms.

**9. Analysis plan**

The ITT and per-protocol effects on the prevention of CVDs and all-cause mortality are estimated in the three age groups. An estimate of the ITT hazard ratio is obtained by fitting a pooled logistic model for the outcome incidence, including the indicators of the assigned strategy (statin initiation at baseline), follow-up period (linear and quadratic terms) and the aforementioned covariates at baseline. In per-protocol analysis, the person-trials are artificially censored when the participant deviated from their assigned strategy unless the patient developed an indication or contraindication for statin therapy, as specified above. To adjust for selection bias resulting from the artificial censoring process described above, each person-trial is weighted at each time point by the inverse probability of receiving their assigned treatment strategy, conditional on the baseline and time-varying covariates:

$${SW}_{m+t}^{A}=\prod_{k=m}^{m+t} \frac{1}{f(A_{k}|\bar{A}_{k-1}, \bar{L}_{k}, \bar{Y}_{k-1}=0)}$$

$A_{k}$: indicator for treatment at month *k*

$\bar{L}_{k}$: covariates history at month *k*

$\bar{Y}_{k-1}$: indicator for outcome of interest at month *k-1*

*m*: indicator for trial *m*

The pooled logistic model is fitted to predict the probability of receiving statin therapy at each time point. We fit the pooled logistic models below separately for *a*=1 and *a*=0 to generate the probabilities based on different prior treatment status (*a*=1 for the person-trials who initiate statin therapy, *a*=0 for those who did not initiate statin therapy).

$$logit\left( \Pr\left[ A_{k}=1 | A_{k-1}=a,L_{0}, \bar{L}_{k}, \bar{Y}_{k-1}=0 \right] \right)=\theta_{0}+\theta_{1}^{T}L_{0}{+\theta}_{2}^{T}L_{k}$$

The first month of each person-trial is also excluded from the weighting model since the inverse probability of being adherent to the assigned treatment was 1 at time 0. Individuals no longer contribute to the weight models above once they deviate from their assigned strategy for the aforementioned allowable clinical reason. To adjust for potential bias arising from the competing events (i.e., death in the present study), each person-trial additionally received a time-varying inverse probability weight of not dying. The weight is derived from a pooled logistic model that included the indicators of treatment arm, the months of follow-up (linear and quadratic terms), the baseline covariates, and the time-varying covariates. The cumulative product of the estimated weights up to each time point is used as the final weight for each person-trial, which is truncated at 10 to avoid the influence of outliers in the estimated weights when estimating the result.

Finally, a pooled logistic model is fitted to estimate the hazard ratio (HR) for the outcomes between continuous statin therapy and never using statins during the follow-up period, which includes the indicators of the assigned treatment strategy, month of follow-up (linear and quadratic terms), and the baseline covariates, with the adjustment of non-stabilized weight mentioned above. Standard errors are obtained from the pooled logistic model fitted in the matched study participants to generate the CI. As the observations at different timepoints within the same person-trial are correlated, the clustered sandwich estimator is adopted to derive the robust standard errors allowing for clustering.

We estimate the absolute risk of outcome incidence by fitting the aforementioned pooled logistic model for the causal effect estimation, incorporating the added product terms between the treatment indicator and time (linear and quadratic terms). The cumulative risk is standardized to the empirical distribution of the confounders at baseline in the entire population. Nonparametric bootstrapping with 500 samples is used to obtain the 95% confidence intervals of the absolute risk difference. For the per-protocol analysis, both the weighting models for obtaining inverse probability weights and the outcome models for absolute risk estimates are included in the bootstrap program. The Number Needed to Treat (NNT) to prevent one additional outcome is calculated based on the estimated 5-year and 10-year risk difference of overall CVD.
