# Supplementary material for: Cardiovascular outcomes and safety associated with statin therapy for primary prevention in older adults with type 2 diabetes: A target trial emulation study
Source: PLoS Med. 2026 Jun 24;23(6):e1005136. doi: 10.1371/journal.pmed.1005136 (PMC13293410; doi:10.1371/journal.pmed.1005136)
Supplement: S2 Appendix — (DOCX) [file pmed.1005136.s002.docx]

**S2 Appendix. Study Protocol**

| Title | Cardiovascular outcomes and safety associated with statin therapy for primary prevention in older adults with type 2 diabetes: a target trial emulation study |
| --- | --- |
| Trial registration | Not applicable |
| Protocol version | 03; Date: 1/18/2026 |
| Funding | National Natural Science Foundation of China (NSFC) Excellent Young Scientists Fund (Hong Kong and Macau) (No. 82222902), Health and Medical Research Fund from Health Bureau of the Government of the Hong Kong SAR (No. 05190107 and 22233101).  (The funder had no role in design, conduct and reporting of the study.) |
| Author details | Linda Chan, MD,  Wanchun Xu, PhD,  Esther W. Chan, PhD,  Eric Yuk Fai Wan, PhD |

**1. BACKGROUND**

Type 2 diabetes (T2D) is a prevalent condition around the world. T2D patients are typically at an elevated risk of developing cardiovascular disease (CVD). As one of the most commonly prescribed lipid-lowering agents, statins are extensively used to mitigate the risk of CVDs in T2D patients with hyperlipidemia. However, there is a lack of consensus regarding the use of statins for primary prevention in T2D patients with advanced age, as limited robust evidence exists on statin use for CVD primary prevention among the older (75-84 years) and very old (≥85 years) T2D patients due to underrepresentation of this population in randomized controlled trials.

**2. OBJECTIVES**

To evaluate the effectiveness and safety of statin use for primary prevention against CVD and all-cause mortality in the old (75-84 years) and very-old (≥85 years) T2D patients with hyperlipidemia.

**3. STUDY DESIGN**

**Design:** target trial emulation.

**Data source:** electronic health records from the clinical management system (CMS) of the Hong Kong Hospital Authority. Reference data for the available information is provided on a monthly basis.

**4. STUDY POPULATION**

**Inclusion criteria:** The analysis will include all T2D patients aged ≥60 years with elevated low-density lipoprotein cholesterol (LDL-C) ≥2.6 mmol/L in each calendar month from January 2009 to December 2015. An LDL-C cut-off of 2.6 mmol/L is chosen as this represents the optimal treatment target for T2D patients with no prior history of CVD according to local guidelines (1).

**Exclusion criteria:** Patients who used statins before baseline, or used fibrates or any other lipid-lowering drugs classified under the British National Formulary code 2.12 before baseline will be excluded from the analysis. Individuals with a history of type 1 diabetes, CVDs, cancer, myopathies or liver dysfunction will be excluded. Patients with incomplete data for the study variables at baseline will also be excluded. Eligible patients need to have at least one follow-up visit record after baseline.

**5. STUDY OUTCOME AND FOLLOW-UP PERIOD**

**Primary outcome:** major CVDs (i.e., a composite outcome of myocardial infarction, heart failure, stroke).

**Secondary outcome:** the three CVD subtypes, all-cause mortality, and muscle-related adverse events (AEs) and liver dysfunction.

After peer review, we also analyzed the composite outcome of major CVD diseases without heart failure as a sensitivity analysis for comparison, and expanded the assessment of muscle-related AEs to include myalgia, myositis, myopathy, and rhabdomyolysis.

Case definition was based on the International Classification of Primary Care, 2nd Edition (ICPC-2) and International Classification of Diseases, 9th Revision, Clinical Modification (ICD-9-CM), or relevant clinical parameters (Appendix I: S3 Table). We excluded patients with pre-existing diagnosed outcomes of interest on or before baseline to ensure the outcome events were restricted to the new incident cases occurring after baseline. The patients with documented old MI diagnosis (ICD-9 code: 412) or late effects of cerebrovascular disease (ICD-9 code: 438.x) on or before baseline were also excluded. For consistency, these two codes were also included in the case definitions for outcome events for MI and stroke. After peer review, we also conducted a sensitivity analysis where these two codes were excluded from the case definition of outcome events for comparison

Patients will be followed up until the outcome of interest, death, or the administrative end of our study (31 December 2018), whichever occurs first. Potential bias of undiagnosed disease might exist for the participants who experienced the outcome incidence within the first year of follow-up. After peer review, we retained these patients in the main analysis and conducted a sensitivity analysis by excluding these patients to test the potential bias (2).

**6. EXPOSURE**

Statin therapy, defined as the treatment with any dose of simvastatin, atorvastatin, fluvastatin, rosuvastatin, lovastatin, pitavastatin, and/or pravastatin.

**7. COVARIATES**

Demographic characteristics (sex and age), clinical parameters plus blood profile (systolic blood pressure, diastolic blood pressure, hemoglobin A1c, LDL-C, high-density lipoprotein cholesterol, total cholesterol, and estimated glomerular filtration rate), comorbidities (hypertension, peripheral vascular disease, atrial fibrillation, chronic obstructive pulmonary disease, renal disease, dementia, obesity, and Charlson Comorbidity Index), drug history within 1 year before baseline (aspirin, insulin, oral antidiabetic drugs, β-blockers, calcium channel blockers, diuretics, and angiotensin-converting enzyme inhibitors), service utilization within the prior 1 year (Specialist Out-Patient Clinic attendance and hospitalization) and lifestyle behavior (smoking status).

**8. ANALYSIS**

The method of target trial emulation (3, 4) will be applied to examine the relationship between statin therapy and risk of the outcomes of interest. The patients will be categorized into different age groups (60-74, 75-84, ≥85 years) for analysis. The analysis of those aged between 60-74 years will serve as a benchmark to test the validity of our emulated trial since the effect of statin therapy is well-established in this population (5, 6).

**Main analysis:** Propensity score matching will be employed to emulate the randomization of the eligible person-trials at baseline, where initiators and non-initiators will be matched in a 1:1 ratio within each age group (60–74, 75–84, ≥85 years). The matching factors included the aforementioned covariates and the baseline calendar month. The intention-to-treat (ITT) and per-protocol effects on the prevention of CVDs and all-cause mortality will be estimated in the emulated target trials in the three age groups. The ITT analysis will compare the risk for outcome incidence between statin initiators and non-initiators, as defined by their treatment strategy at baseline. The hazard ratio will be estimated by fitting a pooled logistic model for the outcome incidence, including the indicators of the assigned strategy (statin initiation at baseline), follow-up period (linear and quadratic terms), and the aforementioned covariates at baseline. The per-protocol analysis will compare risks for the outcomes of interest between continuous users and those who never use statins during the follow-up period, where the person-trials will be artificially censored if participants deviate from their assigned strategy unless they develop an indication or contraindication for statin therapy. To adjust for selection bias resulting from the artificial censoring process, each person-trial will be weighted at each time point by the inverse probability of receiving their assigned treatment strategy, conditional on baseline and time-varying covariates. The pooled logistic model will be fitted to predict the probability of receiving statin therapy at each time point. The last observation carried forward method will be employed to handle missing values in time-varying clinical parameters during the follow-up period. To adjust for potential bias arising from competing events (i.e., death), each person-trial will be additionally adjusted by a time-varying inverse probability weight of not dying. As the treatment strategies in the per-protocol analysis described above are adaptive to the time-varying clinical characteristics (i.e., LDL-C levels and the occurrence of any subtypes of CVDs), we adopted the non-stabilized inverse probability weight, rather than stabilized weight, for estimation of the per-protocol effect after peer review. This change of analytical choice aims to avoid the potential bias introduced by the numerator of stabilized weight. Finally, a pooled logistic model will be fitted to estimate the hazard ratio for outcomes between continuous statin therapy and never using statins during the follow-up period, incorporating the indicators of the assigned treatment strategy, month of follow-up, and baseline covariates, with adjustment for non-stabilized weights as mentioned above. The E-value was calculated to assess the robustness of the estimated results to the potential unmeasured confounding. The absolute risk of the outcome incidence will be estimated by fitting the aforementioned pooled logistic model for causal effect estimation, incorporating product terms between the treatment indicator and time.

**Subgroup analysis:** Subgroup analyses for the risk of overall CVD incidence and all-cause mortality will be conducted based on sex and Charlson Comorbidity Index (<8/≥8) at baseline.

**Sensitivity analysis**: Several sensitivity analyses were conducted. (1) To examine if the length of grace period for the ascertainment of statin discontinuation would have an impact on the results, a sensitivity analysis was conducted by extending this gap from 1 month to 3 months in per-protocol analysis. (2) To evaluate the residual confounding by indication, the patients with familial hypercholesterolemia at baseline were excluded in another sensitivity analysis. (3) An additional sensitivity analysis was conducted regarding the weight truncation at the cut-off of 20. After peer review, we additionally performed the sensitivity analyses below to test the robustness of our results: (4) To test whether the choice of LDL-C cut-off might influence the results, we performed a sensitivity analysis on all patients with T2DM. (5) Regarding the competing event of death, we conducted a sensitivity analysis of estimating the total effects, where death was not considered as a censoring event. (6) As mentioned, a sensitivity analysis was conducted by excluding the participants who had the outcome within the first year of follow-up. (7) As the main analysis was conducted in the matched sample, the estimation better reflects Average Treatment Effect on the Treated (ATT) rather than Average Treatment Effect (ATE). To investigate whether the estimated ATT would differ from the ATE in our study, we additionally performed a sensitivity analysis by including all eligible study participants before matching. (8) Finally, a sensitivity analysis was conducted by removing the exclusion criterion of requiring at least one follow-up visit after baseline. Instead, the person-trials were censored two years after their last recorded visit within the local public healthcare system (i.e., considered them lost to follow-up at this time point) and additionally applied inverse probability weights to account for censoring due to loss to follow-up. (9) For the primary composite outcome, we also analyzed the composite outcome without heart failure for comparison. (10) Finally, for the outcomes of MI and stroke, we performed a sensitivity analysis by excluding the old MI diagnosis (ICD-9 code: 412) or late effects of cerebrovascular disease (ICD-9 code: 438.x) in the case definition for outcome events after baseline.

All analyses will be conducted from September to December 2023 using Stata/MP, version 17.0 (StataCorp LLC). Statistical significance is defined as a two-tailed p-value <0.05.

**9. RELEVANT RESEARCH CHECKLIST**

This study is reported as per TrAnsparent ReportinG of studies Emulating a Target trial (TARGET) guideline.

**REFERENCES**

1. Health Bureau. Hong Kong Reference Framework for Diabetes Care for Adults in Primary Care Settings. 2023.

2. Danaei G, Rodriguez LA, Cantero OF, Logan R, Hernan MA. Observational data for comparative effectiveness research: an emulation of randomised trials of statins and primary prevention of coronary heart disease. Stat Methods Med Res. 2013;22(1):70-96.

3. Hernan MA, Wang W, Leaf DE. Target Trial Emulation A Framework for Causal Inference From Observational Data. Jama-J Am Med Assoc. 2022;328(24):2446-7.

4. Hernan MA, Robins JM. Using Big Data to Emulate a Target Trial When a Randomized Trial Is Not Available. Am J Epidemiol. 2016;183(8):758-64.

5. Cholesterol Treatment Trialists' Collaboration. Efficacy and safety of statin therapy in older people: a meta-analysis of individual participant data from 28 randomised controlled trials. Lancet. 2019;393(10170):407-15.

6. Mangione CM, Barry MJ, Nicholson WK, Cabana M, Chelmow D, Coker TR, et al. Statin Use for the Primary Prevention of Cardiovascular Disease in Adults: US Preventive Services Task Force Recommendation Statement. JAMA. 2022;328(8):746-53.
