# Supplementary material for: Cardiovascular outcomes and safety associated with statin therapy for primary prevention in older adults with type 2 diabetes: A target trial emulation study
Source: PLoS Med. 2026 Jun 24;23(6):e1005136. doi: 10.1371/journal.pmed.1005136 (PMC13293410; doi:10.1371/journal.pmed.1005136)
Supplement: S3 Appendix — Table A - Percentage of missing data for baseline covariates before excluding patients with incomplete information. Table B - Case definitions. Method A - Details on the per-protocol analysis and the estimation of standardized risk differences. Table C - Baseline characteristics of eligible person-trials before matching. Table D - Crude incidence rates of the outcome events (S4a Unadjusted crude incidence rate of the outcome events in the person-trials before matching; S4b Crude incidence rates of the outcome events in the final analytical samples). Table E - E-value of the outcome estimates in the intention-to-treat analysis. Table F - Coefficients of weighting model for treatment history (S6a treatment group; S6b control group). Table G - Estimated standardized 5-year and 10-year absolute risk differences for all outcomes. Table H - Estimated hazard ratios (95% CI) for all-cause mortality, stratified by sex and Charlson Comorbidity Index (CCI). Table I - Sensitivity analysis for using a gap of 3 months for the ascertainment of statin discontinuation in per-protocol analysis: estimated hazard ratio for outcomes of interest. Table J - Sensitivity analysis of truncating the inverse probability weights at 20: estimated hazard ratio for outcomes of interest. Table K - Sensitivity analysis adjusting for excluding the patients with familial hypercholesterolemia: estimated hazard ratio for outcomes of interest. Table L - Sensitivity analysis of including all patients with T2DM (regardless of LDL-C levels): estimated hazard ratio for outcomes of interest. Table M - Sensitivity analysis of estimating the total effect for CVD outcomes and adverse events: estimated hazard ratio for outcomes of interest. Table N - Sensitivity analysis of excluding the participants who had the outcome within the first year of follow-up: estimated hazard ratio for outcomes of interest. Table O - Sensitivity analysis of including all eligible study participants before matching: estimated hazard r [file pmed.1005136.s003.docx]

**Supplementary Information –S3 Appendix**

**Cardiovascular outcomes and safety associated with statin therapy for primary prevention in older adults with type 2 diabetes: a target trial emulation study**

|  | **Content** | **Page** |  |
| --- | --- | --- | --- |
| **Table A** | Percentage of missing data for baseline covariates before excluding patients with incomplete information | 1 |  |
| **Table B** | Case definitions | 2 |  |
| **Method A** | Details on the per-protocol analysis and the estimation of standardized risk differences | 3-4 |  |
| **Table C** | Baseline characteristics of eligible person-trials before matching | 5 |  |
| **Table D** | Crude incidence rates of the outcome events (D-1 Unadjusted crude incidence rate of the outcome events in the person-trials before matching; D-2 Crude incidence rates of the outcome events in the final analytical samples) | 6-7 |  |
| **Table E** | E-value of the outcome estimates in the intention-to-treat analysis | 8 |  |
| **Table F** | Coefficients of weighting model for treatment history (F-1 treatment group; F-2 control group) | 9-10 |  |
| **Table G** | Estimated standardized 5-year and 10-year absolute risk differences for all outcomes | 11 |  |
| **Table H** | Estimated hazard ratios (95% CI) for all-cause mortality, stratified by sex and Charlson Comorbidity Index (CCI) | 12 |  |
| **Table I** | Sensitivity analysis for using a gap of 3 months for the ascertainment of statin discontinuation in per-protocol analysis: estimated hazard ratio for outcomes of interest | 13 |  |
| **Table J** | Sensitivity analysis of truncating the inverse probability weights at 20: estimated hazard ratio for outcomes of interest | 14 |  |
| **Table K** | Sensitivity analysis adjusting for excluding the patients with familial hypercholesterolemia: estimated hazard ratio for outcomes of interest | 15 |  |
| **Table L** | Sensitivity analysis of including all patients with T2DM (regardless of LDL-C levels): estimated hazard ratio for outcomes of interest | 16 |  |
| **Table M** | Sensitivity analysis of estimating the total effect for CVD outcomes and adverse events: estimated hazard ratio for outcomes of interest | 17 |  |
| **Table N** | Sensitivity analysis of excluding the participants who had the outcome within the first year of follow-up: estimated hazard ratio for outcomes of interest | 18 |  |
| **Table O** | Sensitivity analysis of including all eligible study participants before matching: estimated hazard ratio for outcomes of interest | 19 |  |
| **Table P** | Sensitivity analysis of censoring patients two years after their last recorded visit within the local public healthcare system: estimated hazard ratio for outcomes of interest | 20 |  |
| **Table Q** | Sensitivity analysis of the primary composite outcome of major cardiovascular diseases without heart failure | 21 |  |
| **Table R** | Sensitivity analysis of excluding the diagnosis code of non-incident events in case definition of outcome events | 22 |  |
| **Figure A** | Distribution of the propensity score | 23 |  |
| **Figure B** | Distribution of weight distribution by treatment strategies | 24 |  |

**Table A. Percentage of missing data for baseline covariates before excluding patients with incomplete information**

|  | **Percentage missing** | |
| --- | --- | --- |
|  | **Initiators** | **Non-initiators** |
| Age | 0.00% | 0.00% |
| Sex | 0.00% | 0.00% |
| Smoking | 0.00% | 0.00% |
| SBP | 7.64% | 12.79% |
| DBP | 7.64% | 12.79% |
| HbA1c | 2.28% | 3.34% |
| LDL-C | 0.00% | 0.00% |
| HDL-C | 0.01% | 0.03% |
| Total cholesterol | 0.02% | 0.04% |
| eGFR | 0.79% | 1.19% |
| Charlson Comorbidity Index | 0.00% | 0.00% |
| Hypertension | 0.00% | 0.00% |
| Obesity | 0.00% | 0.00% |
| Peripheral vascular disease | 0.00% | 0.00% |
| Atrial fibrillation | 0.00% | 0.00% |
| COPD | 0.00% | 0.00% |
| Renal disease | 0.00% | 0.00% |
| Dementia | 0.00% | 0.00% |
| Long-term aspirin users | 0.00% | 0.00% |
| Insulin | 0.00% | 0.00% |
| Oral antidiabetic drugs | 0.00% | 0.00% |
| ACEI/ARB | 0.00% | 0.00% |
| β-blocker | 0.00% | 0.00% |
| Calcium channel blockers | 0.00% | 0.00% |
| Diuretic | 0.00% | 0.00% |
| Anti-hypertension drugs | 0.00% | 0.00% |
| SOPC attendance in the past 1 year | 0.00% | 0.00% |
| Hospitalization in the past 1 year | 0.00% | 0.00% |

**Table B: Case definitions**

| Events | ICPC-2 | | ICD-9CM | | Clinical indicators |
| --- | --- | --- | --- | --- | --- |
|  | Code | Description | Code | Description |  |
| **Study population** | | | | |  |
| Diabetes | T89 | Diabetes insulin dependent | 250.x | Diabetes mellitus |  |
|  | T90 | Diabetes non-insulin dependent |  |  |  |
| Type 1 diabetes  (exclusion criterion) | T89 | Diabetes insulin dependent | 250.01 | Diabetes mellitus without mention of complication, type I [juvenile type], not stated as uncontrolled |  |
|  |  |  | 250.03 | Diabetes mellitus without mention of complication, type I [juvenile type], uncontrolled |  |
| **Study outcome** | | | | |  |
| Myocardial infarction | K75 | Acute myocardial infarction | 410.x | Acute myocardial infarction |  |
|  |  |  | 411.x | Other acute and subacute forms of ischemic heart disease |  |
|  |  |  | 412 | Old myocardial infarction |  |
| Stroke | K89 | Transient cerebral ischemia | 430 | Subarachnoid hemorrhage |  |
|  | K90 | Stroke/cerebrovascular accident | 431 | Intracerebral hemorrhage |  |
|  | K91 | Cerebrovascular disease | 432.x | Other and unspecified intracranial hemorrhage |  |
|  |  |  | 433.x | Occlusion and stenosis of precerebral arteries |  |
|  |  |  | 434.x | Occlusion of cerebral arteries |  |
|  |  |  | 435.x | Transient cerebral ischemia |  |
|  |  |  | 436 | Acute, but ill-defined, cerebrovascular disease |  |
|  |  |  | 437.x | Other and ill-defined cerebrovascular disease |  |
|  |  |  | 438.x | Late effects of cerebrovascular disease |  |
| Heart failure | K77 | Heart failure | 428.x | Heart failure | Creatine kinase level ≥1600 IU/L |
| Muscle-related disorder | L99 | Musculoskeletal disease | 359.x | Muscular dystrophies and other myopathies |  |
|  |  |  | 729.1 | Myalgia and myositis, unspecified |  |
|  |  |  | 728.88 | Rhabdomyolysis |  |
| Liver dysfunction | D97 | Liver disease NOS | 570.x | Acute and subacute necrosis of liver | ALT ≥ 200U/L or  AST ≥ 80U/L or  ALT ≥ 120U/L & total bilirubin ≥ 38 µmol/L |
|  |  |  | 571.x | Chronic liver disease and cirrhosis |  |
|  |  |  | 572.x | Liver abscess and sequelae of chronic liver disease |  |
|  |  |  | 573.x | Other disorders of liver |  |

**Method A: Details on the per-protocol analysis and the estimation of standardized risk differences**

A time-discrete dataset was constructed for all time-varying variables by month for each eligible person-trial. The table is the sample data for two hypothetical individuals to illustrate the data structure.

| **Unique**  **ID** | **Trial** | **Baseline** | **Eligible** | **Follow-up month** | **Initiator at baseline** | **Treatment strategy** | **Current**  **User** | **Event** |
| --- | --- | --- | --- | --- | --- | --- | --- | --- |
| 1 | 1 | 200901 | 1 | 0 | 0 | 0 | 0 | 0 |
| 1 | 1 | 200901 | 1 | 1 | 0 | 0 | 0 | 0 |
| 1 | 1 | 200901 | 1 | ⁞ | ⁞ | ⁞ | ⁞ | ⁞ |
| 1 | 2 | 200902 | 1 | 0 | 0 | 0 | 0 | 0 |
| 1 | ⁞ | ⁞ | 1 | ⁞ | ⁞ | ⁞ | ⁞ | ⁞ |
| 1 | 36 | 201112 | 1 | 0 | 0 | 0 | 0 | ⁞ |
| 1 | 36 | 201112 | 1 | ⁞ | ⁞ | ⁞ | ⁞ | ⁞ |
| 2 | 13 | 201001 | 1 | 0 | 0 | 0 | 0 | 0 |
| 2 | 13 | 201001 | 1 | 1 | 0 | 0 | 1 | 0 |
| 2 | 13 | 201001 | 1 | ⁞ | ⁞ | ⁞ | ⁞ | ⁞ |
| 2 | ⁞ | ⁞ | 1 | ⁞ | ⁞ | ⁞ | ⁞ | ⁞ |
| 2 | 25 | 201101 | 1 | 0 | 1 | 1 | 1 | 0 |
| 2 | 25 | 201101 | 1 | 1 | 1 | 1 | 1 | 0 |
| 2 | 25 | 201101 | 1 | ⁞ | ⁞ | ⁞ | ⁞ | ⁞ |
| 2 | 25 | 201101 | 1 | 55 | 1 | 1 | 1 | 1 |
| ⁞ | ⁞ | ⁞ | ⁞ | ⁞ | ⁞ | ⁞ | ⁞ | ⁞ |

The statin initiators were censored when they discontinued statin therapy unless the discontinuity was due to the occurrence of adverse events of statin treatment (i.e., myopathies and liver dysfunction). A one-month grace period was given for confirmation of statin discontinuation, where patients were censored one month after the end of their statin prescription if it was not followed by another refill prescription. For the statin non-initiators at baseline, the person-trials were not censored if they started using statins in response to an indication of hyperlipidaemia (the most recent LDL-C ≥2.6 mmol/L; or the most recent LDL-C ≥1.8 mmol/L after the first incidence of coronary heart disease, stroke or heart failure) occurred during the follow-up period, where the information on diseases and LDL-C level were updated by month in the dataset. The rationale of considering the medical allowable reasons in the artificial censoring was based on the emulation of a sensible protocol. For example, it is highly unlikely that a sensible protocol will mandate the statin therapy to be continued when adverse events occurred.

To estimate the non-stabilized inverse probability weight below,

$${SW}_{m+t}^{A}=\prod_{k=m}^{m+t} \frac{1}{f(A_{k}|\bar{A}_{k-1}, \bar{L}_{k}, \bar{Y}_{k-1}=0)}$$

$A_{k}$: indicator for treatment at month *k*

$\bar{L}_{k}$: covariates history at month *k*

$\bar{Y}_{k-1}$: indicator for outcome of interest at month *k-1*

*m*: indicator for trial *m*

To estimate the denominator in the non-stabilized weight above, we fitted the pooled logistic models below separately for *a*=1 and *a*=0 to generate the probabilities based on different prior treatment status (*a*=1 for the person-trials who initiate statin therapy, *a*=0 for those who did not initiate statin therapy).

$$logit\left( \Pr\left[ A_{k}=1 | A_{k-1}=a,L_{0}, \bar{L}_{k}, \bar{Y}_{k-1}=0 \right] \right)=\theta_{0}+\theta_{1}^{T}L_{0}{+\theta}_{2}^{T}L_{k}$$

The first month of each person-trial was also excluded from the weighting model since the inverse probability of being adherent to the assigned treatment was 1 at time 0. Individuals no longer contributed to the weight models adjust for potential bias arising from the competing events (i.e., death in the present study), each person-trial additionally received a time-varying inverse probability weight of not dying. The weight was derived from a pooled logistic model that included the indicators of treatment arm, the months of follow-up (linear and quadratic term), the baseline covariates, and the time-varying covariates. The cumulative product of the estimated weights up to each time point was used as the final weight for each person-trial, which was truncated at 10 to avoid the influence of outliers in the estimated weights when estimating the result. We estimated the absolute risk of outcome incidence by fitting the aforementioned pooled logistic model for the causal effect estimation, incorporating the added product terms between treatment indicator and time (linear and quadratic terms). The cumulative risk was standardized to the empirical distribution of the confounders at baseline in the entire population, which was achieved by averaging the estimates under each treatment strategy at each time point. Nonparametric bootstrapping with 500 samples was used to obtain a 95% confidence interval (CI) of the absolute risk difference.

**Table C.** **Baseline characteristics of eligible person-trials before matching**

|  | **60-74 years** | | | **75-84 years** | | | **≥ 85 years** | | |
| --- | --- | --- | --- | --- | --- | --- | --- | --- | --- |
|  | **Initiator** | **Non-initiator** | **SMD** | **Initiator** | **Non-initiator** | **SMD** | **Initiator** | **Non-initiator** | **SMD** |
|  | 53,240 | 1,464,361 |  | 15,402 | 546,860 |  | 1,899 | 106,990 |  |
| Age | 66.6 (4.2) | 66.9 (4.3) | 0.05 | 79.0 (2.7) | 79.3 (2.7) | 0.10 | 87.8 (2.4) | 88.2 (2.7) | 0.17 |
| Sex (male) | 24,209 (45.5%) | 689,015 (47%) | 0.03 | 5,782 (37.5%) | 208,964 (38%) | 0.01 | 558 (29.4%) | 29,808 (28%) | 0.03 |
| Smoking | 2,394 (4.5%) | 61,886 (4%) | 0.01 | 415 (2.7%) | 14,279 (3%) | <0.01 | 40 (2.1%) | 1,889 (2%) | 0.02 |
| Blood pressure |  |  |  |  |  |  |  |  |  |
| SBP | 148.2 (16.4) | 148.3 (16.4) | <0.01 | 151.9 (16.5) | 152.3 (16.6) | 0.02 | 153.2 (17.2) | 153.0 (17.7) | 0.01 |
| DBP | 81.8 (9.2) | 81.1 (9.2) | 0.07 | 77.9 (9.4) | 77.5 (9.4) | 0.04 | 76.1 (9.5) | 75.3 (9.8) | 0.09 |
| HbA1c | 7.5 (1.6) | 7.4 (1.4) | 0.10 | 7.3 (1.4) | 7.2 (1.3) | 0.05 | 7.0 (1.3) | 7.0 (1.2) | 0.06 |
| Lipid profile |  |  |  |  |  |  |  |  |  |
| LDL-C |  |  |  |  |  |  |  |  |  |
| mmol/L | 3.7 (0.7) | 3.3 (0.6) | 0.67 | 3.6 (0.7) | 3.3 (0.6) | 0.66 | 3.7 (0.7) | 3.3 (0.6) | 0.69 |
| mg/dL | 141.3 (25.6) | 126.7 (21.8) | 0.67 | 140.0 (25.7) | 125.9 (21.4) | 0.66 | 141.8 (25.7) | 126.7 (21.7) | 0.69 |
| HDL-C |  |  |  |  |  |  |  |  |  |
| mmol/L | 1.3 (0.3) | 1.3 (0.3) | 0.09 | 1.4 (0.3) | 1.3 (0.4) | 0.11 | 1.4 (0.4) | 1.4 (0.4) | 0.07 |
| mg/dL | 51.6 (12.9) | 50.5 (13.4) | 0.09 | 52.5 (13.5) | 50.9 (13.8) | 0.11 | 53.3 (14.2) | 52.3 (14.1) | 0.07 |
| Total cholesterol |  |  |  |  |  |  |  |  |  |
| mmol/L | 5.7 (0.8) | 5.2 (0.7) | 0.63 | 5.6 (0.8) | 5.2 (0.7) | 0.62 | 5.7 (0.8) | 5.3 (0.7) | 0.64 |
| mg/dL | 219.2 (30.9) | 202.1 (27.1) | 0.63 | 218.3 (30.6) | 201.6 (26.7) | 0.62 | 221.0 (30.8) | 203.7 (27.2) | 0.64 |
| eGFR | 98.6 (24.3) | 97.9 (24.4) | 0.03 | 82.6 (25.1) | 81.8 (24.7) | 0.03 | 73.8 (23.1) | 73.4 (24.5) | 0.02 |
| Comorbidities |  |  |  |  |  |  |  |  |  |
| Charlson Comorbidity Index | 3.9 (1.6) | 3.9 (1.7) | <0.01 | 5.9 (2.1) | 5.9 (2.1) | <0.01 | 7.3 (2.1) | 7.2 (2.1) | 0.02 |
| Hypertension | 41,527 (78.0%) | 1,111,441 (75.9%) | 0.05 | 13,884 (90.1%) | 484,225 (88.5%) | 0.05 | 1,751 (92.2%) | 98,452 (92.0%) | <0.01 |
| Obesity | 8,911 (16.7%) | 213,913 (14.6%) | 0.06 | 1,911 (12.4%) | 55,311 (10.1%) | 0.07 | 161 (8.5%) | 7,053 (6.6%) | 0.07 |
| Peripheral vascular disease | 156 (0.3%) | 3,636 (0.2%) | <0.01 | 81 (0.5%) | 2,609 (0.5%) | <0.01 | 17 (0.9%) | 781 (0.7%) | 0.02 |
| Atrial fibrillation |  |  |  |  |  |  |  |  |  |
| COPD | 980 (1.8%) | 30,582 (2.1%) | 0.02 | 575 (3.7%) | 22,765 (4.2%) | 0.02 | 100 (5.3%) | 5,692 (5.3%) | <0.01 |
| Renal disease | 8,364 (15.7%) | 230,297 (15.7%) | <0.01 | 5,769 (37.5%) | 202,453 (37.0%) | <0.01 | 999 (52.6%) | 55,178 (51.6%) | 0.02 |
| Dementia | 121 (0.2%) | 4,212 (0.3%) | 0.01 | 264 (1.7%) | 12,016 (2.2%) | 0.03 | 80 (4.2%) | 5,916 (5.5%) | 0.06 |
| Drug use |  |  |  |  |  |  |  |  |  |
| Long-term aspirin users | 2,883 (5.4%) | 65,160 (4.4%) | 0.04 | 1,375 (8.9%) | 43,829 (8.0%) | 0.03 | 220 (11.6%) | 11,504 (10.8%) | 0.03 |
| Insulin | 2,495 (4.7%) | 76,665 (5.2%) | 0.03 | 722 (4.7%) | 28,378 (5.2%) | 0.02 | 84 (4.4%) | 5,063 (4.7%) | 0.01 |
| Oral antidiabetic drugs | 44,429 (83.5%) | 1,224,482 (83.6%) | <0.01 | 12,639 (82.1%) | 457,869 (83.7%) | 0.04 | 1,491 (78.5%) | 85,689 (80.1%) | 0.04 |
| ACEI/ARB | 25,292 (47.5%) | 659,851 (45.1%) | 0.05 | 8,570 (55.6%) | 280,369 (51.3%) | 0.09 | 1,042 (54.9%) | 54,524 (51.0%) | 0.08 |
| β-blocker | 15,423 (29.0%) | 430,693 (29.4%) | <0.01 | 5,326 (34.6%) | 184,173 (33.7%) | 0.02 | 643 (33.9%) | 33,282 (31.1%) | 0.06 |
| Calcium channel blockers | 31,432 (59.0%) | 790,668 (54.0%) | 0.10 | 11,494 (74.6%) | 382,518 (69.9%) | 0.10 | 1,516 (79.8%) | 81,291 (76.0%) | 0.09 |
| Diuretic | 7,003 (13.2%) | 197,353 (13.5%) | <0.01 | 2,798 (18.2%) | 101,382 (18.5%) | <0.01 | 359 (18.9%) | 21,998 (20.6%) | 0.04 |
| Service utilization |  |  |  |  |  |  |  |  |  |
| SOPC attendance in the past 1 year | 26,917 (50.6%) | 809,621 (55.3%) | 0.09 | 9,389 (61.0%) | 351,348 (64.2%) | 0.07 | 1,163 (61.2%) | 68,121 (63.7%) | 0.05 |
| Hospitalization in the past 1 year | 5,555 (10.4%) | 151,574 (10.4%) | <0.01 | 2,653 (17.2%) | 99,831 (18.3%) | 0.03 | 457 (24.1%) | 29,351 (27.4%) | 0.08 |

Notes: LDL-C = Low Density Lipoprotein - Cholesterol; HDL-C = High Density Lipoprotein- cholesterol; TC = Total Cholesterol; ACEI/ARB = angiotensin-converting enzyme inhibitor and angiotensin receptor blocker; SOPC = Specialist Out-patient Clinics

**Table D-1. Unadjusted crude incidence rate of the outcome events in the person-trials before matching**

|  | ITT analysis | | | | | | Per-protocol analysis | | | | | |
| --- | --- | --- | --- | --- | --- | --- | --- | --- | --- | --- | --- | --- |
|  | Treatment group | | | Control group | | | Treatment group | | | Control group | | |
|  | Number of events | Person-year | Incidence rate (per 1,000 person-years) | Number of events | Person-year | Incidence rate (per 1,000 person-years) | Number of events | Person-year | Incidence rate (per 1,000 person-years) | Number of events | Person-year | Incidence rate (per 1,000 person-years) |
| **60-74 years old** |  |  |  |  |  |  |  |  |  |  |  |  |
| Overall CVD | 5216 | 305,971 | 17.05 (16.59, 17.52) | 179951 | 9,212,553 | 19.53 (19.44, 19.62) | 4146 | 255,057 | 16.26 (15.77, 16.76) | 103935 | 5,138,021 | 20.23 (20.11, 20.35) |
| Myocardial infarction | 1384 | 317,039 | 4.37 (4.14, 4.60) | 48928 | 9,623,461 | 5.08 (5.04, 5.13) | 1067 | 263,414 | 4.05 (3.81, 4.30) | 27742 | 5,311,021 | 5.22 (5.16, 5.29) |
| Heart failure | 1466 | 316,905 | 4.63 (4.40, 4.87) | 49226 | 9,627,881 | 5.11 (5.07, 5.16) | 1151 | 263,391 | 4.37 (4.12, 4.63) | 24662 | 5,297,218 | 4.66 (4.60, 4.71) |
| Stroke | 3214 | 311,185 | 10.33 (9.98, 10.69) | 112642 | 9,396,758 | 11.99 (11.92, 12.06) | 2558 | 259,009 | 9.88 (9.50, 10.27) | 66188 | 5,200,539 | 12.73 (12.63, 12.82) |
| Death | 3617 | 320,326 | 11.29 (10.93, 11.67) | 132644 | 9,747,770 | 13.61 (13.53, 13.68) | 2545 | 265,917 | 9.57 (9.21, 9.95) | 69460 | 5,343,568 | 13.00 (12.90, 13.10) |
| Muscle related AE | 754 | 318,741 | 2.37 (2.20, 2.54) | 25355 | 9,689,547 | 2.62 (2.58, 2.65) | 592 | 264,593 | 2.24 (2.06, 2.43) | 13650 | 5,322,312 | 2.56 (2.52, 2.61) |
| Liver dysfunction | 3571 | 311,660 | 11.46 (11.09, 11.84) | 119551 | 9,444,302 | 12.66 (12.59, 12.73) | 2761 | 258,845 | 10.67 (10.28, 11.07) | 66213 | 5,215,141 | 12.70 (12.60, 12.79) |
| **75-84 years old** |  |  |  |  |  |  |  |  |  |  |  |  |
| Overall CVD | 3492 | 78,302 | 44.60 (43.14, 46.10) | 147536 | 2,906,395 | 50.76 (50.50, 51.02) | 2803 | 65,325 | 42.91 (41.35, 44.53) | 101355 | 1,945,216 | 52.10 (51.78, 52.43) |
| Myocardial infarction | 928 | 85,087 | 10.91 (10.23, 11.63) | 41922 | 3,197,490 | 13.11 (12.99, 13.24) | 735 | 70,561 | 10.42 (9.69, 11.20) | 28445 | 2,103,374 | 13.52 (13.37, 13.68) |
| Heart failure | 1363 | 83,864 | 16.25 (15.41, 17.14) | 58954 | 3,145,579 | 18.74 (18.59, 18.89) | 1114 | 69,543 | 16.02 (15.11, 16.99) | 38116 | 2,061,379 | 18.49 (18.31, 18.68) |
| Stroke | 1951 | 81,717 | 23.88 (22.84, 24.96) | 81542 | 3,056,279 | 26.68 (26.50, 26.86) | 1528 | 68,000 | 22.47 (21.37, 23.63) | 55398 | 2,020,942 | 27.41 (27.18, 27.64) |
| Death | 3234 | 86,745 | 37.28 (36.02, 38.59) | 149203 | 3,272,264 | 45.60 (45.37, 45.83) | 2291 | 71,812 | 31.90 (30.62, 33.24) | 96680 | 2,130,272 | 45.38 (45.10, 45.67) |
| Muscle related AE | 344 | 86,187 | 3.99 (3.59, 4.44) | 13217 | 3,248,654 | 4.07 (4.00, 4.14) | 272 | 71,340 | 3.81 (3.39, 4.29) | 8508 | 2,119,160 | 4.01 (3.93, 4.10) |
| Liver dysfunction | 1627 | 83,852 | 19.40 (18.48, 20.37) | 65925 | 3,152,615 | 20.91 (20.75, 21.07) | 1288 | 69,353 | 18.57 (17.58, 19.61) | 41899 | 2,065,467 | 20.29 (20.09, 20.48) |
| **≥85 years old** |  |  |  |  |  |  |  |  |  |  |  |  |
| Overall CVD | 539 | 8,028 | 67.14 (61.71, 73.05) | 35842 | 436,327 | 82.14 (81.30, 83.00) | 458 | 6,690 | 68.46 (62.47, 75.02) | 30793 | 358,346 | 85.93 (84.98, 86.90) |
| Myocardial infarction | 163 | 8,811 | 18.50 (15.87, 21.57) | 10715 | 490,451 | 21.85 (21.44, 22.26) | 131 | 7,306 | 17.93 (15.11, 21.28) | 8555 | 398,583 | 21.46 (21.01, 21.92) |
| Heart failure | 224 | 8,636 | 25.94 (22.75, 29.57) | 16379 | 475,692 | 34.43 (33.91, 34.96) | 189 | 7,173 | 26.35 (22.85, 30.39) | 13870 | 385,225 | 36.00 (35.41, 36.61) |
| Stroke | 273 | 8,470 | 32.23 (28.63, 36.29) | 18366 | 466,393 | 39.38 (38.81, 39.95) | 233 | 7,034 | 33.13 (29.14, 37.67) | 15907 | 380,746 | 41.78 (41.13, 42.43) |
| Death | 699 | 9,007 | 77.60 (72.06, 83.58) | 51566 | 503,628 | 102.39 (101.51, 103.28) | 515 | 7,460 | 69.04 (63.33, 75.27) | 41376 | 406,476 | 101.79 (100.82, 102.78) |
| Muscle related AE | 44 | 8,967 | 4.91 (3.65, 6.59) | 1960 | 500,813 | 3.91 (3.74, 4.09) | 36 | 7,422 | 4.85 (3.50, 6.72) | 1387 | 404,579 | 3.43 (3.25, 3.61) |
| Liver dysfunction | 230 | 8,707 | 26.42 (23.21, 30.06) | 13961 | 485,356 | 28.76 (28.29, 29.25) | 177 | 7,217 | 24.52 (21.17, 28.42) | 11444 | 393,095 | 29.11 (28.58, 29.65) |

**Table D-2: Crude incidence rates of the outcome events in the final analytical samples**

|  | ITT analysis | | | | | | Per-protocol analysis | | | | | |
| --- | --- | --- | --- | --- | --- | --- | --- | --- | --- | --- | --- | --- |
|  | Treatment group | | | Control group | | | Treatment group | | | Control group | | |
|  | Number of event | Person-year | Incidence rate (per 1,000 person-years) | Number of event | Person-year | Incidence rate (per 1,000 person-years) | Number of event | Person-year | Incidence rate (per 1,000 person-years) | Number of event | Person-year | Incidence rate (per 1,000 person-years) |
| **60-74 years old** |  |  |  |  |  |  |  |  |  |  |  |  |
| Overall CVD | 5216 | 305,971 | 17.05 (16.59, 17.52) | 5829 | 299,041 | 19.49 (19.00, 20.00) | 4146 | 255,057 | 16.26 (15.77, 16.76) | 3122 | 156,537 | 19.94 (19.26, 20.66) |
| Myocardial infarction | 1384 | 317,039 | 4.37 (4.14, 4.60) | 1614 | 310,706 | 5.19 (4.95, 5.45) | 1067 | 263,414 | 4.05 (3.81, 4.30) | 833 | 161,145 | 5.17 (4.83, 5.53) |
| Heart failure | 1466 | 316,905 | 4.63 (4.40, 4.87) | 1552 | 310,698 | 5.00 (4.75, 5.25) | 1151 | 263,391 | 4.37 (4.12, 4.63) | 724 | 160,925 | 4.50 (4.18, 4.84) |
| Stroke | 3214 | 311,185 | 10.33 (9.98, 10.69) | 3602 | 304,586 | 11.83 (11.45, 12.22) | 2558 | 259,009 | 9.88 (9.50, 10.27) | 1977 | 158,334 | 12.49 (11.95, 13.05) |
| Death | 3617 | 320,326 | 11.29 (10.93, 11.67) | 3855 | 314,292 | 12.27 (11.88, 12.66) | 2545 | 265,917 | 9.57 (9.21, 9.95) | 1915 | 162,170 | 11.81 (11.29, 12.35) |
| Muscle related AE | 754 | 318,741 | 2.37 (2.20, 2.54) | 831 | 312,516 | 2.66 (2.48, 2.85) | 592 | 264,593 | 2.24 (2.06, 2.43) | 420 | 161,568 | 2.60 (2.36, 2.86) |
| Liver dysfunction | 3571 | 311,660 | 11.46 (11.09, 11.84) | 3909 | 305,276 | 12.80 (12.41, 13.21) | 2761 | 258,845 | 10.67 (10.28, 11.07) | 2043 | 158,748 | 12.87 (12.32, 13.44) |
| **75-84 years old** |  |  |  |  |  |  |  |  |  |  |  |  |
| Overall CVD | 3492 | 78,302 | 44.60 (43.14, 46.10) | 3739 | 75,916 | 49.25 (47.70, 50.86) | 2803 | 65,325 | 42.91 (41.35, 44.53) | 2445 | 47,063 | 51.95 (49.93, 54.05) |
| Myocardial infarction | 928 | 85,087 | 10.91 (10.23, 11.63) | 1111 | 82,687 | 13.44 (12.67, 14.25) | 735 | 70,561 | 10.42 (9.69, 11.20) | 723 | 50,701 | 14.26 (13.26, 15.34) |
| Heart failure | 1363 | 83,864 | 16.25 (15.41, 17.14) | 1468 | 81,505 | 18.01 (17.11, 18.96) | 1114 | 69,543 | 16.02 (15.11, 16.99) | 920 | 49,834 | 18.46 (17.31, 19.69) |
| Stroke | 1951 | 81,717 | 23.88 (22.84, 24.96) | 2060 | 79,329 | 25.97 (24.87, 27.11) | 1528 | 68,000 | 22.47 (21.37, 23.63) | 1320 | 48,742 | 27.08 (25.66, 28.58) |
| Death | 3234 | 86,745 | 37.28 (36.02, 38.59) | 3585 | 84,439 | 42.46 (41.09, 43.87) | 2291 | 71,812 | 31.90 (30.62, 33.24) | 2182 | 51,364 | 42.48 (40.74, 44.30) |
| Muscle related AE | 344 | 86,187 | 3.99 (3.59, 4.44) | 343 | 83,949 | 4.09 (3.68, 4.54) | 272 | 71,340 | 3.81 (3.39, 4.29) | 213 | 51,152 | 4.16 (3.64, 4.76) |
| Liver dysfunction | 1627 | 83,852 | 19.40 (18.48, 20.37) | 1634 | 81,685 | 20.00 (19.06, 21.00) | 1288 | 69,353 | 18.57 (17.58, 19.61) | 1005 | 49,917 | 20.13 (18.93, 21.42) |
| **≥85 years old** |  |  |  |  |  |  |  |  |  |  |  |  |
| Overall CVD | 539 | 8,028 | 67.14 (61.71, 73.05) | 576 | 7,601 | 75.78 (69.84, 82.23) | 458 | 6,690 | 68.46 (62.47, 75.02) | 472 | 5,879 | 80.28 (73.36, 87.86) |
| Myocardial infarction | 163 | 8,811 | 18.50 (15.87, 21.57) | 170 | 8,369 | 20.31 (17.48, 23.61) | 131 | 7,306 | 17.93 (15.11, 21.28) | 128 | 6,445 | 19.86 (16.70, 23.62) |
| Heart failure | 224 | 8,636 | 25.94 (22.75, 29.57) | 232 | 8,253 | 28.11 (24.72, 31.97) | 189 | 7,173 | 26.35 (22.85, 30.39) | 189 | 6,325 | 29.88 (25.91, 34.46) |
| Stroke | 273 | 8,470 | 32.23 (28.63, 36.29) | 298 | 8,012 | 37.19 (33.20, 41.67) | 233 | 7,034 | 33.13 (29.14, 37.67) | 253 | 6,164 | 41.05 (36.29, 46.43) |
| Death | 699 | 9,007 | 77.60 (72.06, 83.58) | 800 | 8,587 | 93.16 (86.92, 99.85) | 515 | 7,460 | 69.04 (63.33, 75.27) | 614 | 6,577 | 93.36 (86.26, 101.04) |
| Muscle related AE | 44 | 8,967 | 4.91 (3.65, 6.59) | 33 | 8,558 | 3.86 (2.74, 5.42) | 36 | 7,422 | 4.85 (3.50, 6.72) | 18 | 6,560 | 2.74 (1.73, 4.36) |
| Liver dysfunction | 230 | 8,707 | 26.42 (23.21, 30.06) | 245 | 8,292 | 29.55 (26.07, 33.49) | 177 | 7,217 | 24.52 (21.17, 28.42) | 185 | 6,376 | 29.02 (25.12, 33.51) |

Notes: The crude incidence rates in the per-protocol analysis refer to incidence rate before the adjustment

**Table E. E-value of the outcome estimates in the intention-to-treat analysis**

|  | **E-value** |
| --- | --- |
| **Age 60-74 years** |  |
| Overall CVD | 1.60 |
| Myocardial infarction | 1.70 |
| Heart failure | 1.46 |
| Stroke | 1.60 |
| Death | 1.46 |
| Muscle related AE | 1.50 |
| Liver dysfunction | 1.50 |
| **Age 60-74 years** |  |
| Overall CVD | 1.53 |
| Myocardial infarction | 1.88 |
| Heart failure | 1.56 |
| Stroke | 1.43 |
| Death | 1.67 |
| Muscle related AE | 1.25 |
| Liver dysfunction | 1.29 |
| **Age 60-74 years** |  |
| Overall CVD | 1.60 |
| Myocardial infarction | 1.56 |
| Heart failure | 1.39 |
| Stroke | 1.70 |
| Death | 1.88 |
| Muscle related AE | 2.13 |
| Liver dysfunction | 1.53 |

**Table F-1. Coefficients of weighting model for treatment history (treatment group)**

|  | **Age 60-74 years** | | **Age 75-84 years** | | **Age ≥85 years** | |
| --- | --- | --- | --- | --- | --- | --- |
| **Variable** | **Coef** | **STD error** | **Coef** | **STD error** | **Coef** | **STD error** |
| **Baseline variables** |  |  |  |  |  |  |
| Sex, male | 0.1881* | 0.0328 | 0.1712* | 0.0610 | 0.1063 | 0.1884 |
| Age, year | -0.0170* | 0.0046 | -0.0073 | 0.0131 | -0.0024 | 0.0297 |
| Smoking status | 0.0339 | 0.1571 | -0.2261 | 0.3320 | 0.8957 | 1.5134 |
| HbA1c | -0.1127* | 0.0100 | -0.1036* | 0.0209 | -0.1043 | 0.0747 |
| SBP | -0.0021 | 0.0012 | -0.0052* | 0.0019 | -0.0093 | 0.0052 |
| DBP | -0.0008 | 0.0021 | 0.0005 | 0.0034 | 0.0122 | 0.0093 |
| LDL-C | 0.5486* | 0.0745 | 0.5041* | 0.1133 | 0.8363* | 0.3504 |
| HDL-C | -0.4341* | 0.0766 | -0.6512* | 0.1220 | -0.3006 | 0.3494 |
| Total cholesterol | 0.2962* | 0.0679 | 0.3426* | 0.1015 | 0.0387 | 0.3239 |
| eGFR | 0.0027* | 0.0009 | 0.0051* | 0.0018 | 0.0144* | 0.0063 |
| Charlson index | 0.1241* | 0.0331 | -0.0279 | 0.0589 | 0.0538 | 0.1827 |
| Hypertension | 0.0387 | 0.0655 | -0.3568* | 0.1427 | 0.0099 | 0.4160 |
| Obesity | 0.0508 | 0.0464 | 0.1571 | 0.0948 | -0.1783 | 0.3084 |
| Peripheral vascular disease | -0.1270 | 0.4698 | -0.8424 | 0.6811 | 1.5811 | 1.1565 |
| Atrial fibrillation | -0.1694 | 0.2246 | 0.3686 | 0.2540 | 2.9862* | 1.1550 |
| COPD | -0.2429 | 0.1841 | 0.2387 | 0.2605 | -0.2005 | 0.5750 |
| Renal disease | -0.4487* | 0.1470 | 0.0867 | 0.2533 | 0.1941 | 0.7900 |
| Dementia | 0.2529 | 0.3911 | 0.1588 | 0.2413 | -0.1586 | 0.4778 |
| ACEI/ARB user | -0.3600* | 0.0409 | -0.3840* | 0.0671 | -0.4897* | 0.1822 |
| β-blocker user | -0.3164* | 0.0486 | -0.3171* | 0.0753 | 0.0247 | 0.2034 |
| Calcium channel blockers user | -0.5429* | 0.0463 | -0.4864* | 0.0835 | -0.6003* | 0.2229 |
| Diuretic user | -0.0966 | 0.0528 | -0.1048 | 0.0824 | -0.4427* | 0.1993 |
| Oral diabetic drug user | -1.6765* | 0.0605 | -1.2281* | 0.0912 | -0.9352* | 0.2137 |
| Insulin user | -0.0424 | 0.0898 | 0.1384 | 0.1704 | 1.1100 | 0.5912 |
| Aspirin user | -0.3383* | 0.0975 | -0.1363 | 0.1316 | -0.5862 | 0.3569 |
| Specialist Out-patient Visit | -0.0539 | 0.0343 | 0.0313 | 0.0617 | -0.1123 | 0.1691 |
| Hospitalization | -0.1146* | 0.0518 | -0.0441 | 0.0753 | -0.0766 | 0.1910 |
| **Time-varying variable** |  |  |  |  |  |  |
| Smoking status | -0.0362 | 0.1417 | 0.4716 | 0.2980 | -1.1756 | 1.4217 |
| HbA1c | 0.0484* | 0.0115 | 0.0510* | 0.0215 | 0.0566 | 0.0666 |
| SBP | -0.0054* | 0.0008 | -0.0039* | 0.0012 | -0.0037 | 0.0033 |
| DBP | -0.0010 | 0.0014 | -0.0013 | 0.0022 | -0.0019 | 0.0058 |
| LDL-C | -0.7057* | 0.0593 | -0.7278* | 0.1048 | -0.5288 | 0.3264 |
| HDL-C | 0.9204* | 0.0774 | 0.8346* | 0.1274 | 0.7078* | 0.3467 |
| Total cholesterol | -0.5346* | 0.0547 | -0.5376* | 0.0959 | -0.6255* | 0.2957 |
| eGFR | -0.0006 | 0.0007 | -0.0028* | 0.0010 | -0.0083* | 0.0040 |
| Charlson index | -0.0772* | 0.0137 | -0.0615* | 0.0258 | -0.0788 | 0.0690 |
| Hypertension | -0.2860* | 0.0632 | 0.0972 | 0.1538 | -0.0307 | 0.4504 |
| Obesity | 0.0741* | 0.0304 | 0.1005 | 0.0569 | 0.2132 | 0.1624 |
| Peripheral vascular disease | -0.2376 | 0.3551 | 0.8664 | 0.5853 | -0.4779 | 0.7864 |
| Atrial fibrillation | -0.1430 | 0.1439 | -0.5166* | 0.1577 | -0.4159 | 0.3991 |
| COPD | 0.2076 | 0.1477 | -0.1363 | 0.2109 | -0.0674 | 0.4179 |
| Renal disease | 0.3559* | 0.0731 | 0.3149* | 0.1322 | 0.0909 | 0.3572 |
| Dementia | -0.3849* | 0.1775 | -0.1638 | 0.1401 | 0.1794 | 0.3261 |
| ACEI/ARB user | 0.9178* | 0.0367 | 0.9697* | 0.0577 | 1.3196* | 0.1656 |
| β-blocker user | 0.5698* | 0.0483 | 0.5412* | 0.0776 | 0.4983* | 0.2115 |
| Calcium channel blockers user | 1.0676* | 0.0388 | 1.2014* | 0.0605 | 1.1108* | 0.1665 |
| Diuretic user | 0.3740* | 0.0554 | 0.3024* | 0.0832 | 0.2906 | 0.2075 |
| Insulin user | 0.1977* | 0.0696 | -0.0806 | 0.1272 | -0.2155 | 0.2597 |
| Aspirin user | 0.6700* | 0.0812 | 0.4864* | 0.1056 | 0.5361 | 0.2987 |
| Oral diabetic drug user | 2.4894* | 0.0459 | 1.7937* | 0.0699 | 1.3774* | 0.1650 |
| Specialist Out-patient Visit | 0.0680* | 0.0268 | 0.1897* | 0.0465 | 0.3942* | 0.1250 |
| Hospitalization | -0.0368 | 0.0236 | -0.0572 | 0.0349 | -0.0894 | 0.0967 |
| Month | -0.0594* | 0.0011 | -0.0614* | 0.0022 | -0.0588* | 0.0061 |
| Month square | 0.0005* | <0.0001 | 0.0005* | <0.0001 | 0.0004* | 0.0001 |
| Intercept | 5.1166* | 0.3734 | 5.4580* | 1.0264 | 4.2467 | 3.1251 |

**Table F-2. Coefficients of weighting model for treatment history (control group)**

|  | **Age 60-74 years** | | **Age 75-84 years** | | **Age ≥85 years** | |
| --- | --- | --- | --- | --- | --- | --- |
| **Variable** | **Coef** | **STD error** | **Coef** | **STD error** | **Coef** | **STD error** |
| **Baseline variables** |  |  |  |  |  |  |
| Sex, male | -0.0298 | 0.0387 | -0.1728* | 0.0801 | 0.2873 | 0.1961 |
| Age, year | -0.0214* | 0.0051 | -0.0413* | 0.0158 | -0.0724* | 0.0334 |
| Smoking status | -0.6275* | 0.1986 | 0.3400 | 0.5124 | 0.2427 | 0.5486 |
| HbA1c | -0.1325* | 0.0164 | -0.0902* | 0.0327 | 0.0304 | 0.1283 |
| SBP | -0.0033* | 0.0015 | -0.0013 | 0.0026 | -0.0069 | 0.0044 |
| DBP | 0.0019 | 0.0024 | -0.0051 | 0.0044 | 0.0122 | 0.0090 |
| LDL-C | 0.2867* | 0.0591 | 0.3574 | 0.1897 | 0.2585 | 0.3505 |
| HDL-C | -0.8091* | 0.0974 | -1.0383* | 0.1840 | -0.7255 | 0.4037 |
| Total cholesterol | 0.7197* | 0.0551 | 0.6680* | 0.1420 | 0.6821* | 0.3259 |
| eGFR | 0.0001 | 0.0012 | -0.0026 | 0.0022 | 0.0040 | 0.0054 |
| Charlson index | 0.0911* | 0.0385 | -0.0946 | 0.0663 | -0.1670 | 0.1390 |
| Hypertension | -0.1456 | 0.0938 | -0.0380 | 0.1945 | -1.0955* | 0.4557 |
| Obesity | 0.1564* | 0.0501 | 0.1084 | 0.1113 | -0.3194 | 0.2033 |
| Peripheral vascular disease | -0.8320 | 0.4365 | 0.5238 | 0.7983 | 1.7143* | 0.8390 |
| Atrial fibrillation | -0.2221 | 0.2766 | 0.0014 | 0.4231 | 0.2512 | 0.6206 |
| COPD | 0.0397 | 0.2913 | 0.1265 | 0.5480 | -0.4239 | 0.7684 |
| Renal disease | -0.5966* | 0.1716 | 0.0431 | 0.2782 | 1.3787* | 0.6366 |
| Dementia | 0.0426 | 0.3768 | -0.9083* | 0.2621 | -0.8977 | 0.5385 |
| ACEI/ARB user | -0.2224* | 0.0495 | -0.3709* | 0.0958 | -0.4545* | 0.1360 |
| β-blocker user | -0.4281* | 0.0611 | -0.2411* | 0.1132 | 0.5238 | 0.3934 |
| Calcium channel blockers user | -0.1328* | 0.0608 | -0.2031 | 0.1131 | -0.6015* | 0.2870 |
| Diuretic user | -0.1697* | 0.0633 | -0.2662* | 0.1152 | -0.3224 | 0.1879 |
| Oral diabetic drug user | -0.6686* | 0.0695 | -0.4963* | 0.1696 | -1.0773* | 0.1990 |
| Insulin user | -0.2072 | 0.1101 | -0.0418 | 0.2662 | -0.3346 | 0.3772 |
| Aspirin user | -0.8051* | 0.1127 | -0.7497* | 0.1833 | -1.5888* | 0.2748 |
| Specialist Out-patient Visit | -0.0317 | 0.0452 | -0.1116 | 0.0819 | -0.1292 | 0.2293 |
| Hospitalization | -0.1673* | 0.0660 | -0.1116 | 0.1132 | -0.1132 | 0.2014 |
| **Time-varying variable** |  |  |  |  |  |  |
| Smoking status | 0.5971* | 0.1806 | -0.3352 | 0.4908 | / | / |
| HbA1c | 0.0887* | 0.0144 | 0.1119* | 0.0257 | 0.0196 | 0.0951 |
| SBP | -0.0033* | 0.0009 | -0.0017 | 0.0019 | -0.0088* | 0.0034 |
| DBP | -0.0045* | 0.0016 | 0.0090* | 0.0029 | 0.0113 | 0.0064 |
| LDL-C | -0.3923* | 0.0612 | -0.6305* | 0.1367 | -0.1199 | 0.3140 |
| HDL-C | 0.7057* | 0.0843 | 0.8497* | 0.1442 | 0.7027 | 0.3726 |
| Total cholesterol | -0.5532* | 0.0554 | -0.5166* | 0.1148 | -0.6623* | 0.2573 |
| eGFR | -0.0009 | 0.0009 | -0.0002 | 0.0010 | -0.0093* | 0.0034 |
| Charlson index | -0.0331 | 0.0232 | 0.1274* | 0.0399 | 0.1382* | 0.0483 |
| Hypertension | 0.0682 | 0.0932 | -0.0128 | 0.1971 | -0.2430 | 0.6655 |
| Obesity | 0.1069* | 0.0365 | 0.0754 | 0.0695 | 0.4099* | 0.1634 |
| Peripheral vascular disease | 0.6655* | 0.3255 | 0.2409 | 0.6780 | -1.8181* | 0.6365 |
| Atrial fibrillation | 0.1665 | 0.1669 | 0.2230 | 0.2762 | -0.0890 | 0.3304 |
| COPD | -0.2240 | 0.2572 | -0.6905 | 0.5185 | -0.3234 | 0.6757 |
| Renal disease | 0.2198 | 0.1140 | -0.3513 | 0.1825 | -0.9873* | 0.2486 |
| Dementia | -0.7266* | 0.2593 | 0.2790 | 0.2215 | 0.2249 | 0.4368 |
| ACEI/ARB user | 0.3865* | 0.0454 | 0.6500* | 0.0893 | 0.6590* | 0.1391 |
| β-blocker user | 0.4030* | 0.0574 | 0.3377* | 0.1036 | -0.1491 | 0.3721 |
| Calcium channel blockers user | 0.3349* | 0.0551 | 0.3508* | 0.0954 | 0.8218* | 0.2991 |
| Diuretic user | 0.0377 | 0.0592 | 0.3633* | 0.1060 | 0.2677 | 0.1532 |
| Insulin user | 0.1781 | 0.0917 | 0.3143 | 0.1829 | 0.1330 | 0.1994 |
| Aspirin user | 1.3038* | 0.0811 | 0.9101* | 0.1383 | 2.1207* | 0.2387 |
| Oral diabetic drug user | 0.9711* | 0.0666 | 0.6773* | 0.1510 | 1.1121* | 0.1986 |
| Specialist Out-patient Visit | 0.0283 | 0.0374 | 0.0075 | 0.0658 | 0.3054 | 0.1773 |
| Hospitalization | 0.0464 | 0.0282 | 0.0014 | 0.0490 | -0.2378 | 0.1523 |
| Month | -0.0090* | 0.0012 | -0.0225* | 0.0027 | -0.0430* | 0.0073 |
| Month square | 0.0001* | <0.0001 | 0.0002* | <0.0001 | 0.0005* | 0.0001 |
| Intercept | -1.4614* | 0.4174 | -0.0166 | 1.2637 | 3.0563 | 2.6772 |

Notes: * p<0.05; / omitted because of collinearity

**Table G: Estimated standardized 5-year and 10-year absolute risk differences for all outcomes**

|  | **ITT analysis** | | | | |  |  | **Per-protocol analysis** | | | | | |  |  |
| --- | --- | --- | --- | --- | --- | --- | --- | --- | --- | --- | --- | --- | --- | --- | --- |
|  | **Number of events** | | 5-year risk difference | p-value | 10-year risk difference | p-value |  | **Number of events** | | 5-year risk difference | p-value | 10-year risk difference | p-value | |  |
|  | Initiators | Non-initiators |  |  |  |  |  | Initiators | Non-initiators |  |  |  |  |  |  |
| **Aged 60-74 years** |  |  |  |  |  |  |  |  |  |  |  |  |  | |  |
| Overall CVD | 5216 | 5829 | -1.2% (-1.5%, -0.9%) | p<0.001 | -1.6% (-2.6%, -0.7%) | 0.001 |  | 4146 | 3122 | -2.3% (-2.9%, -1.7%) | p<0.001 | -5.6% (-8.1%, -3.1%) | p<0.001 | |  |
| MI | 1384 | 1614 | -0.4% (-0.5%, -0.2%) | p<0.001 | -0.9% (-1.4%, -0.3%) | 0.001 |  | 1067 | 833 | -0.7% (-1.1%, -0.4%) | p<0.001 | -1.6% (-2.9%, -0.2%) | 0.023 | |  |
| Heart failure | 1466 | 1552 | -0.2% (-0.4%, 0.0%) | 0.017 | -0.5% (-1.1%, 0.0%) | 0.062 |  | 1151 | 724 | -0.4% (-0.7%, -0.1%) | 0.020 | -2.3% (-4.1%, -0.5%) | 0.013 | |  |
| Stroke | 3214 | 3602 | -0.8% (-1.0%, -0.5%) | p<0.001 | -1.1% (-1.9%, -0.2%) | 0.018 |  | 2558 | 1977 | -1.4% (-1.9%, -0.9%) | p<0.001 | -2.9% (-5.1%, -0.7%) | 0.009 | |  |
| Death | 3617 | 3855 | -0.5% (-0.8%, -0.3%) | p<0.001 | -1.2% (-2.1%, -0.4%) | 0.005 |  | 2545 | 1915 | -1.4% (-1.9%, -1.0%) | p<0.001 | -4.8% (-6.8%, -2.7%) | p<0.001 | |  |
| Muscle AE | 754 | 831 | -0.1% (-0.3%, 0.0%) | 0.061 | -0.4% (-0.8%, 0.1%) | 0.094 |  | 592 | 420 | -0.1% (-0.4%, 0.1%) | 0.290 | -0.4% (-1.3%, 0.6%) | 0.466 | |  |
| Liver dysfunction | 3571 | 3909 | -0.6% (-0.9%, -0.4%) | p<0.001 | -1.1% (-2.0%, -0.2%) | 0.018 |  | 2761 | 2043 | -1.5% (-2.0%, -1.0%) | p<0.001 | -1.3% (-2.9%, 0.3%) | 0.115 | |  |
| **Aged 75-84 years** |  |  |  |  |  |  |  |  |  |  |  |  |  | |  |
| Overall CVD | 3492 | 3739 | -2.5% (-3.4%, -1.6%) | p<0.001 | -1.6% (-4.0%, 0.9%) | 0.220 |  | 2803 | 2445 | -6.6% (-8.2%, -4.9%) | p<0.001 | -8.2% (-13.2%, -3.3%) | 0.001 | |  |
| MI | 928 | 1111 | -1.4% (-1.9%, -0.8%) | p<0.001 | -2.2% (-3.8%, -0.5%) | 0.009 |  | 735 | 723 | -2.3% (-3.4%, -1.3%) | p<0.001 | -4.9% (-8.2%, -1.5%) | 0.005 | |  |
| Heart failure | 1363 | 1468 | -1.2% (-1.8%, -0.6%) | p<0.001 | -0.4% (-2.5%, 1.7%) | 0.702 |  | 1114 | 920 | -3.2% (-4.4%, -2.1%) | p<0.001 | -4.1% (-8.5%, 0.2%) | 0.061 | |  |
| Stroke | 1951 | 2060 | -1.2% (-2.0%, -0.5%) | 0.001 | -0.5% (-2.6%, 1.7%) | 0.666 |  | 1528 | 1320 | -3.2% (-4.5%, -1.9%) | p<0.001 | -3.6% (-7.9%, 0.7%) | 0.103 | |  |
| Death | 3234 | 3585 | -2.7% (-3.4%, -2.0%) | p<0.001 | -2.6% (-5.1%, -0.1%) | 0.038 |  | 2291 | 2182 | -5.4% (-6.8%, -4.0%) | p<0.001 | -12.5% (-17.3%, -7.7%) | p<0.001 | |  |
| Muscle AE | 344 | 343 | -0.1% (-0.4%, 0.3%) | 0.677 | -0.1% (-1.1%, 0.8%) | 0.797 |  | 272 | 213 | -0.1% (-0.7%, 0.5%) | 0.753 | -0.5% (-2.3%, 1.3%) | 0.569 | |  |
| Liver dysfunction | 1627 | 1634 | -0.6% (-1.3%, 0.1%) | 0.088 | 0.4% (-1.9%, 2.8%) | 0.732 |  | 1288 | 1005 | -1.3% (-2.5%, -0.1%) | 0.033 | -1.0% (-5.4%, 3.4%) | 0.662 | |  |
| **Aged ≥85 years** |  |  |  |  |  |  |  |  |  |  |  |  |  | |  |
| Overall CVD | 539 | 576 | -4.3% (-7.3%, -1.3%) | 0.004 | -0.2% (-9.2%, 8.9%) | 0.974 |  | 458 | 472 | -11.4% (-17.3%, -5.5%) | p<0.001 | -7.0% (-20.9%, 6.9%) | 0.326 | |  |
| MI | 163 | 170 | -1.3% (-3.5%, 0.9%) | 0.236 | -0.7% (-7.6%, 6.1%) | 0.831 |  | 131 | 128 | -3.2% (-7.7%, 1.2%) | 0.157 | -3.2% (-16.7%, 10.2%) | 0.636 | |  |
| Heart failure | 224 | 232 | -1.4% (-3.8%, 0.9%) | 0.232 | 1.2% (-8.2%, 10.5%) | 0.805 |  | 189 | 189 | -4.8% (-9.3%, -0.3%) | 0.035 | 5.9% (-9.6%, 21.5%) | 0.456 | |  |
| Stroke | 273 | 298 | -2.9% (-5.5%, -0.3%) | 0.027 | -0.5% (-8.3%, 7.3%) | 0.896 |  | 233 | 253 | -6.4% (-11.3%, -1.5%) | 0.011 | -6.7% (-20.8%, 7.4%) | 0.351 | |  |
| Death | 699 | 800 | -6.3% (-9.2%, -3.4%) | p<0.001 | -7.3% (-15.1%, 0.5%) | 0.065 |  | 515 | 614 | -11.5% (-16.7%, -6.3%) | p<0.001 | -13.4% (-26.7%, -0.2%) | 0.047 | |  |
| Muscle AE | 44 | 33 | 0.9% (-1.2%, 3.0%) | 0.398 | 2.7% (-6.3%, 11.8%) | 0.554 |  | 36 | 18 | 1.8% (-1.7%, 5.4%) | 0.309 | -0.5% (-33.5%, 32.4%) | 0.974 | |  |
| Liver dysfunction | 230 | 245 | -1.8% (-4.3%, 0.7%) | 0.160 | 0.8% (-10.3%, 11.9%) | 0.889 |  | 177 | 185 | -3.3% (-7.7%, 1.0%) | 0.134 | -6.4% (-24.0%, 11.1%) | 0.472 | |  |

Analyses adjusted for sex, age, smoking status, hemoglobin A1c, low-density lipoprotein cholesterol, high-density lipoprotein cholesterol, total cholesterol, estimated glomerular filtration rate, comorbidities (including Charlson Comorbidity Index, hypertension, peripheral vascular disease, chronic obstructive pulmonary disease, atrial fibrillation, renal disease, dementia, obesity), drug use (aspirin, insulin, oral antidiabetic drugs, angiotensin-converting enzyme (ACE) inhibitors, β-blockers, calcium channel blockers, diuretics), Specialist Out-Patient Clinic attendance (within 1 year before baseline) and hospitalization (within 1 year before baseline), baseline calendar month, month of follow-up and its square term, with the added product terms between treatment and time (linear and quadratic term).

**Table H: Estimated hazard ratios (95% CI) for all-cause mortality, stratified by sex and Charlson Comorbidity Index (CCI)**

|  |  |  | **60-74 years old** | | **75-84 years old** | | **≥85 years old** | |
| --- | --- | --- | --- | --- | --- | --- | --- | --- |
|  |  |  | **Hazard ratio**  **(95% CI)** | **p-value** | **Hazard ratio**  **(95% CI)** | **p-value** | **Hazard ratio**  **(95% CI)** | **p-value** |
| **ITT analysis** | **Sex** | Female | 0.88 (0.82, 0.95) | p<0.001 | 0.84 (0.79, 0.90) | p<0.001 | 0.79 (0.69, 0.90) | p<0.001 |
|  |  | Male | 0.91 (0.86, 0.97) | 0.002 | 0.84 (0.78, 0.90) | p<0.001 | 0.78 (0.65, 0.93) | 0.007 |
|  | **CCI** | <8 | 0.91 (0.86, 0.95) | p<0.001 | 0.86 (0.81, 0.92) | p<0.001 | 0.71 (0.60, 0.83) | p<0.001 |
|  |  | ≥8 | 0.86 (0.77, 0.97) | 0.011 | 0.82 (0.76, 0.88) | p<0.001 | 0.85 (0.74, 0.98) | 0.029 |
| **Per-protocol analysis** | **Gender** | Female | 0.64 (0.57, 0.72) | p<0.001 | 0.63 (0.57, 0.70) | p<0.001 | 0.62 (0.51, 0.75) | p<0.001 |
|  |  | Male | 0.80 (0.73, 0.88) | p<0.001 | 0.70 (0.63, 0.78) | p<0.001 | 0.68 (0.51, 0.89) | 0.006 |
|  | **CCI** | <8 | 0.76 (0.69, 0.82) | p<0.001 | 0.67 (0.61, 0.74) | p<0.001 | 0.54 (0.42, 0.68) | p<0.001 |
|  |  | ≥8 | 0.61 (0.51, 0.73) | p<0.001 | 0.65 (0.58, 0.72) | p<0.001 | 0.73 (0.59, 0.90) | 0.003 |

Analyses adjusted for sex, age, smoking status, hemoglobin A1c, low-density lipoprotein cholesterol, high-density lipoprotein cholesterol, total cholesterol, estimated glomerular filtration rate, comorbidities (including Charlson Comorbidity Index, hypertension, peripheral vascular disease, chronic obstructive pulmonary disease, atrial fibrillation, renal disease, dementia, obesity), drug use (aspirin, insulin, oral antidiabetic drugs, angiotensin-converting enzyme (ACE) inhibitors, β-blockers, calcium channel blockers, diuretics), baseline calendar month, month of follow-up and its square term, Specialist Out-Patient Clinic attendance (within 1 year before baseline) and hospitalization (within 1 year before baseline).

**Table I: Sensitivity analysis for using a gap of 3 months for the ascertainment of statin discontinuation in per-protocol analysis: estimated hazard ratio for outcomes of interest**

|  | **Hazard ratio** | **95% CI** | **p-value** |
| --- | --- | --- | --- |
| **Aged 60-74 years** |  |  |  |
| Overall CVD | 0.73 | (0.68, 0.77) | p<0.001 |
| Myocardial infarction | 0.71 | (0.62, 0.80) | p<0.001 |
| Heart failure | 0.77 | (0.68, 0.88) | p<0.001 |
| Stroke | 0.74 | (0.69, 0.81) | p<0.001 |
| Death | 0.70 | (0.65, 0.76) | p<0.001 |
| Muscle related AE | 0.86 | (0.73, 1.03) | 0.098 |
| Liver dysfunction | 0.78 | (0.72, 0.84) | p<0.001 |
| **Aged 75-84 years** |  |  |  |
| Overall CVD | 0.70 | (0.65, 0.75) | p<0.001 |
| Myocardial infarction | 0.64 | (0.55, 0.73) | p<0.001 |
| Heart failure | 0.67 | (0.60, 0.75) | p<0.001 |
| Stroke | 0.76 | (0.69, 0.84) | p<0.001 |
| Death | 0.64 | (0.59, 0.69) | p<0.001 |
| Muscle related AE | 0.90 | (0.70, 1.15) | 0.400 |
| Liver dysfunction | 0.90 | (0.81, 1.00) | 0.056 |
| **Aged ≥ 85 years** |  |  |  |
| Overall CVD | 0.64 | (0.54, 0.76) | p<0.001 |
| Myocardial infarction | 0.71 | (0.51, 0.99) | 0.042 |
| Heart failure | 0.74 | (0.57, 0.97) | 0.030 |
| Stroke | 0.67 | (0.52, 0.87) | 0.002 |
| Death | 0.60 | (0.51, 0.71) | p<0.001 |
| Muscle related AE | 1.79 | (0.81, 3.95) | 0.147 |
| Liver dysfunction | 0.81 | (0.62, 1.06) | 0.130 |

Analyses adjusted for sex, age, smoking status, hemoglobin A1c, low-density lipoprotein cholesterol, high-density lipoprotein cholesterol, total cholesterol, estimated glomerular filtration rate, comorbidities (including Charlson Comorbidity Index, hypertension, peripheral vascular disease, chronic obstructive pulmonary disease, atrial fibrillation, renal disease, dementia, obesity), drug use (aspirin, insulin, oral antidiabetic drugs, angiotensin-converting enzyme (ACE) inhibitors, β-blockers, calcium channel blockers, diuretics), baseline calendar month, month of follow-up and its square term, Specialist Out-Patient Clinic attendance (within 1 year before baseline) and hospitalization (within 1 year before baseline).

**Table J: Sensitivity analysis of truncating the inverse probability weights at 20: estimated hazard ratio for outcomes of interest**

|  | **Hazard ratio** | **95% CI** | **p-value** |
| --- | --- | --- | --- |
| **Aged 60-74 years** |  |  |  |
| Overall CVD | 0.72 | (0.67, 0.77) | p<0.001 |
| Myocardial infarction | 0.69 | (0.60, 0.80) | p<0.001 |
| Heart failure | 0.72 | (0.62, 0.84) | p<0.001 |
| Stroke | 0.75 | (0.68, 0.83) | p<0.001 |
| Death | 0.73 | (0.67, 0.80) | p<0.001 |
| Muscle related AE | 0.86 | (0.70, 1.05) | 0.135 |
| Liver dysfunction | 0.77 | (0.71, 0.85) | p<0.001 |
| **Aged 75-84 years** |  |  |  |
| Overall CVD | 0.67 | (0.62, 0.73) | p<0.001 |
| Myocardial infarction | 0.62 | (0.53, 0.73) | p<0.001 |
| Heart failure | 0.63 | (0.55, 0.72) | p<0.001 |
| Stroke | 0.76 | (0.68, 0.86) | p<0.001 |
| Death | 0.65 | (0.59, 0.70) | p<0.001 |
| Muscle related AE | 0.91 | (0.69, 1.21) | 0.520 |
| Liver dysfunction | 0.89 | (0.78, 1.01) | 0.069 |
| **Aged ≥ 85 years** |  |  |  |
| Overall CVD | 0.62 | (0.50, 0.76) | p<0.001 |
| Myocardial infarction | 0.69 | (0.48, 1.00) | 0.048 |
| Heart failure | 0.73 | (0.53, 1.00) | 0.050 |
| Stroke | 0.69 | (0.52, 0.93) | 0.015 |
| Death | 0.62 | (0.51, 0.74) | p<0.001 |
| Muscle related AE | 1.90 | (0.62, 5.88) | 0.263 |
| Liver dysfunction | 0.81 | (0.59, 1.11) | 0.196 |

Analyses adjusted for sex, age, smoking status, hemoglobin A1c, low-density lipoprotein cholesterol, high-density lipoprotein cholesterol, total cholesterol, estimated glomerular filtration rate, comorbidities (including Charlson Comorbidity Index, hypertension, peripheral vascular disease, chronic obstructive pulmonary disease, atrial fibrillation, renal disease, dementia, obesity), drug use (aspirin, insulin, oral antidiabetic drugs, angiotensin-converting enzyme (ACE) inhibitors, β-blockers, calcium channel blockers, diuretics), baseline calendar month, month of follow-up and its square term, Specialist Out-Patient Clinic attendance (within 1 year before baseline) and hospitalization (within 1 year before baseline).

**Table K: Sensitivity analysis adjusting for excluding the patients with familial hypercholesterolemia: estimated hazard ratio for outcomes of interest**

|  | **ITT analysis** | | | **Per-protocol analysis** | | |
| --- | --- | --- | --- | --- | --- | --- |
|  | **Hazard ratio** | **95% CI** | **p-value** | **Hazard ratio** | **95% CI** | **p-value** |
| **Aged 60-74 years** |  |  |  |  |  |  |
| Overall CVD | 0.87 | (0.83, 0.90) | p<0.001 | 0.73 | (0.69, 0.78) | p<0.001 |
| Myocardial infarction | 0.83 | (0.77, 0.89) | p<0.001 | 0.70 | (0.62, 0.79) | p<0.001 |
| Heart failure | 0.91 | (0.85, 0.98) | 0.014 | 0.81 | (0.71, 0.92) | 0.002 |
| Stroke | 0.86 | (0.82, 0.91) | p<0.001 | 0.75 | (0.69, 0.81) | p<0.001 |
| Death | 0.90 | (0.86, 0.95) | p<0.001 | 0.72 | (0.67, 0.78) | p<0.001 |
| Muscle related AE | 0.88 | (0.80, 0.97) | 0.013 | 0.87 | (0.73, 1.03) | 0.103 |
| Liver dysfunction | 0.89 | (0.85, 0.94) | p<0.001 | 0.78 | (0.73, 0.85) | p<0.001 |
| **Aged 75-84 years** |  |  |  |  |  |  |
| Overall CVD | 0.89 | (0.85, 0.93) | p<0.001 | 0.70 | (0.65, 0.75) | p<0.001 |
| Myocardial infarction | 0.78 | (0.72, 0.86) | p<0.001 | 0.64 | (0.56, 0.73) | p<0.001 |
| Heart failure | 0.87 | (0.81, 0.94) | p<0.001 | 0.68 | (0.60, 0.76) | p<0.001 |
| Stroke | 0.91 | (0.85, 0.97) | 0.003 | 0.77 | (0.70, 0.85) | p<0.001 |
| Death | 0.84 | (0.80, 0.88) | p<0.001 | 0.65 | (0.61, 0.70) | p<0.001 |
| Muscle related AE | 0.97 | (0.83, 1.13) | 0.667 | 0.89 | (0.70, 1.14) | 0.354 |
| Liver dysfunction | 0.96 | (0.89, 1.02) | 0.194 | 0.89 | (0.80, 1.00) | 0.043 |
| **Aged ≥ 85 years** |  |  |  |  |  |  |
| Overall CVD | 0.86 | (0.76, 0.97) | 0.016 | 0.64 | (0.54, 0.77) | p<0.001 |
| Myocardial infarction | 0.87 | (0.69, 1.09) | 0.222 | 0.71 | (0.51, 0.99) | 0.043 |
| Heart failure | 0.92 | (0.76, 1.11) | 0.371 | 0.75 | (0.58, 0.98) | 0.034 |
| Stroke | 0.83 | (0.70, 0.99) | 0.034 | 0.66 | (0.52, 0.85) | p<0.001 |
| Death | 0.78 | (0.70, 0.87) | p<0.001 | 0.61 | (0.52, 0.71) | p<0.001 |
| Muscle related AE | 1.38 | (0.86, 2.21) | 0.186 | 1.78 | (0.78, 4.05) | 0.167 |
| Liver dysfunction | 0.88 | (0.73, 1.06) | 0.178 | 0.79 | (0.60, 1.04) | 0.096 |

Analyses adjusted for sex, age, smoking status, hemoglobin A1c, low-density lipoprotein cholesterol, high-density lipoprotein cholesterol, total cholesterol, estimated glomerular filtration rate, comorbidities (including Charlson Comorbidity Index, hypertension, peripheral vascular disease, chronic obstructive pulmonary disease, atrial fibrillation, renal disease, dementia, obesity), drug use (aspirin, insulin, oral antidiabetic drugs, angiotensin-converting enzyme (ACE) inhibitors, β-blockers, calcium channel blockers, diuretics), baseline calendar month, month of follow-up and its square term, Specialist Out-Patient Clinic attendance (within 1 year before baseline) and hospitalization (within 1 year before baseline).

**Table L. Sensitivity analysis of including all patients with T2DM (regardless of LDL-C levels): estimated hazard ratio for outcomes of interest**

|  | **ITT analysis** | | | **Per-protocol analysis** | | |
| --- | --- | --- | --- | --- | --- | --- |
|  | **Hazard ratio** | **95% CI** | **p-value** | **Hazard ratio** | **95% CI** | **p-value** |
| **Aged 60-74 years** |  |  |  |  |  |  |
| Overall CVD | 0.87 | (0.84, 0.91) | p<0.001 | 0.74 | (0.69, 0.78) | p<0.001 |
| Myocardial infarction | 0.82 | (0.77, 0.88) | p<0.001 | 0.72 | (0.64, 0.81) | p<0.001 |
| Heart failure | 0.96 | (0.90, 1.03) | 0.280 | 0.85 | (0.75, 0.96) | 0.010 |
| Stroke | 0.86 | (0.82, 0.90) | p<0.001 | 0.72 | (0.67, 0.77) | p<0.001 |
| Death | 0.84 | (0.81, 0.88) | p<0.001 | 0.65 | (0.60, 0.69) | p<0.001 |
| Muscle related AE | 0.85 | (0.77, 0.93) | p<0.001 | 0.89 | (0.75, 1.04) | 0.145 |
| Liver dysfunction | 0.90 | (0.86, 0.94) | p<0.001 | 0.83 | (0.77, 0.90) | p<0.001 |
| **Aged 75-84 years** |  |  |  |  |  |  |
| Overall CVD | 0.92 | (0.88, 0.96) | p<0.001 | 0.72 | (0.67, 0.77) | p<0.001 |
| Myocardial infarction | 0.82 | (0.75, 0.89) | p<0.001 | 0.67 | (0.59, 0.76) | p<0.001 |
| Heart failure | 0.90 | (0.84, 0.97) | 0.004 | 0.72 | (0.65, 0.80) | p<0.001 |
| Stroke | 0.94 | (0.88, 1.00) | 0.045 | 0.76 | (0.69, 0.84) | p<0.001 |
| Death | 0.84 | (0.80, 0.88) | p<0.001 | 0.64 | (0.59, 0.69) | p<0.001 |
| Muscle related AE | 0.85 | (0.74, 0.98) | 0.025 | 0.79 | (0.63, 0.99) | 0.039 |
| Liver dysfunction | 0.93 | (0.87, 0.99) | 0.027 | 0.84 | (0.76, 0.93) | 0.001 |
| **Aged ≥ 85 years** |  |  |  |  |  |  |
| Overall CVD | 0.84 | (0.75, 0.95) | 0.005 | 0.66 | (0.56, 0.77) | p<0.001 |
| Myocardial infarction | 0.74 | (0.60, 0.91) | 0.005 | 0.57 | (0.42, 0.77) | p<0.001 |
| Heart failure | 0.79 | (0.66, 0.95) | 0.011 | 0.54 | (0.42, 0.68) | p<0.001 |
| Stroke | 0.79 | (0.68, 0.93) | 0.005 | 0.71 | (0.57, 0.88) | 0.002 |
| Death | 0.76 | (0.69, 0.84) | p<0.001 | 0.63 | (0.54, 0.73) | p<0.001 |
| Muscle related AE | 1.03 | (0.64, 1.67) | 0.898 | 0.63 | (0.36, 1.11) | 0.110 |
| Liver dysfunction | 0.89 | (0.74, 1.06) | 0.194 | 0.65 | (0.51, 0.82) | p<0.001 |

Analyses adjusted for sex, age, smoking status, hemoglobin A1c, low-density lipoprotein cholesterol, high-density lipoprotein cholesterol, total cholesterol, estimated glomerular filtration rate, comorbidities (including Charlson Comorbidity Index, hypertension, peripheral vascular disease, chronic obstructive pulmonary disease, atrial fibrillation, renal disease, dementia, obesity), drug use (aspirin, insulin, oral antidiabetic drugs, angiotensin-converting enzyme (ACE) inhibitors, β-blockers, calcium channel blockers, diuretics), baseline calendar month, month of follow-up and its square term, Specialist Out-Patient Clinic attendance (within 1 year before baseline) and hospitalization (within 1 year before baseline).

**Table M. Sensitivity analysis of estimating the total effect for CVD outcomes and adverse events: estimated hazard ratio for outcomes of interest**

|  | **ITT analysis** | | | **Per-protocol analysis** | | |
| --- | --- | --- | --- | --- | --- | --- |
|  | Hazard ratio | 95% CI | p-value | Hazard ratio | 95% CI | p-value |
| **Aged 60-74 years** |  |  |  |  |  |  |
| Overall CVD | 0.87 | (0.84, 0.90) | p<0.001 | 0.77 | (0.72, 0.82) | p<0.001 |
| Myocardial infarction | 0.84 | (0.78, 0.90) | p<0.001 | 0.77 | (0.68, 0.87) | p<0.001 |
| Heart failure | 0.91 | (0.85, 0.98) | 0.011 | 0.85 | (0.75, 0.96) | 0.012 |
| Stroke | 0.87 | (0.83, 0.91) | p<0.001 | 0.78 | (0.72, 0.85) | p<0.001 |
| Muscle related AE | 0.89 | (0.81, 0.99) | 0.027 | 0.91 | (0.77, 1.08) | 0.294 |
| Liver dysfunction | 0.90 | (0.86, 0.94) | p<0.001 | 0.85 | (0.79, 0.92) | p<0.001 |
| **Aged 75-84 years** |  |  |  |  |  |  |
| Overall CVD | 0.90 | (0.86, 0.94) | p<0.001 | 0.79 | (0.73, 0.85) | p<0.001 |
| Myocardial infarction | 0.80 | (0.73, 0.87) | p<0.001 | 0.75 | (0.65, 0.86) | p<0.001 |
| Heart failure | 0.89 | (0.83, 0.96) | 0.002 | 0.79 | (0.70, 0.88) | p<0.001 |
| Stroke | 0.92 | (0.87, 0.98) | 0.012 | 0.85 | (0.77, 0.94) | 0.002 |
| Muscle related AE | 0.99 | (0.85, 1.15) | 0.930 | 1.06 | (0.83, 1.36) | 0.632 |
| Liver dysfunction | 0.97 | (0.91, 1.04) | 0.413 | 1.06 | (0.95, 1.19) | 0.266 |
| **Aged ≥ 85 years** |  |  |  |  |  |  |
| Overall CVD | 0.91 | (0.80, 1.03) | 0.125 | 0.79 | (0.67, 0.95) | 0.010 |
| Myocardial infarction | 0.92 | (0.73, 1.15) | 0.445 | 1.02 | (0.72, 1.44) | 0.911 |
| Heart failure | 0.96 | (0.80, 1.16) | 0.692 | 0.92 | (0.70, 1.21) | 0.535 |
| Stroke | 0.87 | (0.74, 1.03) | 0.109 | 0.82 | (0.64, 1.05) | 0.109 |
| Muscle related AE | 1.43 | (0.89, 2.29) | 0.137 | 3.00 | (1.25, 7.23) | 0.014 |
| Liver dysfunction | 0.91 | (0.76, 1.10) | 0.342 | 1.03 | (0.78, 1.37) | 0.819 |

Analyses adjusted for sex, age, smoking status, hemoglobin A1c, low-density lipoprotein cholesterol, high-density lipoprotein cholesterol, total cholesterol, estimated glomerular filtration rate, comorbidities (including Charlson Comorbidity Index, hypertension, peripheral vascular disease, chronic obstructive pulmonary disease, atrial fibrillation, renal disease, dementia, obesity), drug use (aspirin, insulin, oral antidiabetic drugs, angiotensin-converting enzyme (ACE) inhibitors, β-blockers, calcium channel blockers, diuretics), baseline calendar month, month of follow-up and its square term, Specialist Out-Patient Clinic attendance (within 1 year before baseline) and hospitalization (within 1 year before baseline).

**Table N. Sensitivity analysis of excluding the participants who had the outcome within the first year of follow-up: estimated hazard ratio for outcomes of interest**

|  | **ITT analysis** | | | **Per-protocol analysis** | | |
| --- | --- | --- | --- | --- | --- | --- |
|  | Hazard ratio | 95% CI | p-value | Hazard ratio | 95% CI | p-value |
| **Aged 60-74 years** |  |  |  |  |  |  |
| Overall CVD | 0.85 | (0.82, 0.89) | p<0.001 | 0.72 | (0.67, 0.77) | p<0.001 |
| Myocardial infarction | 0.81 | (0.75, 0.87) | p<0.001 | 0.70 | (0.61, 0.80) | p<0.001 |
| Heart failure | 0.92 | (0.85, 0.99) | 0.036 | 0.79 | (0.69, 0.90) | p<0.001 |
| Stroke | 0.85 | (0.81, 0.90) | p<0.001 | 0.73 | (0.67, 0.80) | p<0.001 |
| Death | 0.91 | (0.87, 0.96) | p<0.001 | 0.70 | (0.65, 0.76) | p<0.001 |
| Muscle related AE | 0.88 | (0.79, 0.98) | 0.016 | 0.86 | (0.71, 1.04) | 0.112 |
| Liver dysfunction | 0.88 | (0.84, 0.93) | p<0.001 | 0.76 | (0.70, 0.83) | p<0.001 |
| **Aged 75-84 years** |  |  |  |  |  |  |
| Overall CVD | 0.87 | (0.83, 0.92) | p<0.001 | 0.69 | (0.64, 0.75) | p<0.001 |
| Myocardial infarction | 0.75 | (0.68, 0.83) | p<0.001 | 0.62 | (0.54, 0.72) | p<0.001 |
| Heart failure | 0.87 | (0.80, 0.94) | p<0.001 | 0.67 | (0.60, 0.76) | p<0.001 |
| Stroke | 0.90 | (0.84, 0.96) | 0.003 | 0.77 | (0.69, 0.85) | p<0.001 |
| Death | 0.84 | (0.80, 0.89) | p<0.001 | 0.64 | (0.60, 0.70) | p<0.001 |
| Muscle related AE | 0.95 | (0.80, 1.12) | 0.509 | 0.89 | (0.69, 1.16) | 0.385 |
| Liver dysfunction | 0.97 | (0.90, 1.04) | 0.410 | 0.91 | (0.81, 1.02) | 0.104 |
| **Aged ≥85 years** |  |  |  |  |  |  |
| Overall CVD | 0.84 | (0.73, 0.96) | 0.013 | 0.66 | (0.54, 0.80) | p<0.001 |
| Myocardial infarction | 0.88 | (0.68, 1.14) | 0.330 | 0.77 | (0.54, 1.09) | 0.142 |
| Heart failure | 0.91 | (0.74, 1.13) | 0.408 | 0.75 | (0.56, 1.01) | 0.057 |
| Stroke | 0.83 | (0.68, 1.01) | 0.056 | 0.66 | (0.50, 0.87) | 0.003 |
| Death | 0.78 | (0.69, 0.87) | p<0.001 | 0.60 | (0.51, 0.71) | p<0.001 |
| Muscle related AE | 1.26 | (0.76, 2.08) | 0.378 | 1.79 | (0.71, 4.53) | 0.216 |
| Liver dysfunction | 0.86 | (0.70, 1.07) | 0.172 | 0.80 | (0.60, 1.08) | 0.153 |

Analyses adjusted for sex, age, smoking status, hemoglobin A1c, low-density lipoprotein cholesterol, high-density lipoprotein cholesterol, total cholesterol, estimated glomerular filtration rate, comorbidities (including Charlson Comorbidity Index, hypertension, peripheral vascular disease, chronic obstructive pulmonary disease, atrial fibrillation, renal disease, dementia, obesity), drug use (aspirin, insulin, oral antidiabetic drugs, angiotensin-converting enzyme (ACE) inhibitors, β-blockers, calcium channel blockers, diuretics), baseline calendar month, month of follow-up and its square term, Specialist Out-Patient Clinic attendance (within 1 year before baseline) and hospitalization (within 1 year before baseline).

**Table O. Sensitivity analysis of including all eligible study participants before matching: estimated hazard ratio for outcomes of interest**

|  | **ITT analysis** | | | **Per-protocol analysis** | | |
| --- | --- | --- | --- | --- | --- | --- |
|  | Hazard ratio | 95% CI | p-value | Hazard ratio | 95% CI | p-value |
| **Aged 60-74 years** |  |  |  |  |  |  |
| Overall CVD | 0.88 | (0.85, 0.90) | p<0.001 | 0.72 | (0.69, 0.75) | p<0.001 |
| Myocardial infarction | 0.83 | (0.79, 0.88) | p<0.001 | 0.67 | (0.62, 0.73) | p<0.001 |
| Heart failure | 0.92 | (0.87, 0.97) | 0.003 | 0.74 | (0.69, 0.80) | p<0.001 |
| Stroke | 0.88 | (0.84, 0.91) | p<0.001 | 0.74 | (0.70, 0.77) | p<0.001 |
| Death | 0.88 | (0.85, 0.91) | p<0.001 | 0.64 | (0.61, 0.67) | p<0.001 |
| Muscle related AE | 0.88 | (0.82, 0.95) | p<0.001 | 0.82 | (0.74, 0.91) | p<0.001 |
| Liver dysfunction | 0.91 | (0.88, 0.94) | p<0.001 | 0.79 | (0.75, 0.83) | p<0.001 |
| **Aged 75-84 years** |  |  |  |  |  |  |
| Overall CVD | 0.90 | (0.87, 0.93) | p<0.001 | 0.71 | (0.67, 0.74) | p<0.001 |
| Myocardial infarction | 0.82 | (0.77, 0.87) | p<0.001 | 0.67 | (0.61, 0.74) | p<0.001 |
| Heart failure | 0.89 | (0.84, 0.94) | p<0.001 | 0.72 | (0.67, 0.78) | p<0.001 |
| Stroke | 0.93 | (0.88, 0.97) | p<0.001 | 0.75 | (0.70, 0.80) | p<0.001 |
| Death | 0.87 | (0.84, 0.90) | p<0.001 | 0.65 | (0.62, 0.68) | p<0.001 |
| Muscle related AE | 0.95 | (0.85, 1.05) | 0.321 | 0.85 | (0.73, 1.00) | 0.046 |
| Liver dysfunction | 0.95 | (0.90, 1.00) | 0.042 | 0.84 | (0.79, 0.90) | p<0.001 |
| **Aged ≥85 years** |  |  |  |  |  |  |
| Overall CVD | 0.83 | (0.76, 0.91) | p<0.001 | 0.68 | (0.60, 0.77) | p<0.001 |
| Myocardial infarction | 0.79 | (0.67, 0.92) | 0.003 | 0.62 | (0.50, 0.78) | p<0.001 |
| Heart failure | 0.79 | (0.69, 0.90) | p<0.001 | 0.66 | (0.55, 0.80) | p<0.001 |
| Stroke | 0.82 | (0.73, 0.93) | 0.001 | 0.72 | (0.61, 0.85) | p<0.001 |
| Death | 0.80 | (0.74, 0.86) | p<0.001 | 0.62 | (0.56, 0.70) | p<0.001 |
| Muscle related AE | 1.17 | (0.87, 1.59) | 0.301 | 1.20 | (0.79, 1.82) | 0.395 |
| Liver dysfunction | 0.93 | (0.82, 1.06) | 0.293 | 0.79 | (0.65, 0.94) | 0.010 |

Analyses adjusted for sex, age, smoking status, hemoglobin A1c, low-density lipoprotein cholesterol, high-density lipoprotein cholesterol, total cholesterol, estimated glomerular filtration rate, comorbidities (including Charlson Comorbidity Index, hypertension, peripheral vascular disease, chronic obstructive pulmonary disease, atrial fibrillation, renal disease, dementia, obesity), drug use (aspirin, insulin, oral antidiabetic drugs, angiotensin-converting enzyme (ACE) inhibitors, β-blockers, calcium channel blockers, diuretics), baseline calendar month, month of follow-up and its square term, Specialist Out-Patient Clinic attendance (within 1 year before baseline) and hospitalization (within 1 year before baseline).

**Table P. Sensitivity analysis of censoring patients two years after their last recorded visit within the local public healthcare system: estimated hazard ratio for outcomes of interest**

|  | **ITT analysis** | | | **Per-protocol analysis** | | |
| --- | --- | --- | --- | --- | --- | --- |
|  | Hazard ratio | 95% CI | p-value | Hazard ratio | 95% CI | p-value |
| **Aged 60-74 years** |  |  |  |  |  |  |
| Overall CVD | 0.87 | (0.83, 0.90) | p<0.001 | 0.77 | (0.74, 0.81) | p<0.001 |
| Myocardial infarction | 0.81 | (0.76, 0.88) | p<0.001 | 0.74 | (0.67, 0.81) | p<0.001 |
| Heart failure | 0.96 | (0.89, 1.03) | 0.277 | 0.95 | (0.86, 1.05) | 0.283 |
| Stroke | 0.85 | (0.81, 0.89) | p<0.001 | 0.74 | (0.69, 0.79) | p<0.001 |
| Death | 0.85 | (0.81, 0.89) | p<0.001 | 0.61 | (0.57, 0.65) | p<0.001 |
| Muscle related AE | 0.88 | (0.80, 0.97) | 0.013 | 0.88 | (0.77, 1.00) | 0.057 |
| Liver dysfunction | 0.88 | (0.84, 0.92) | p<0.001 | 0.81 | (0.76, 0.87) | p<0.001 |
| **Aged 75-84 years** |  |  |  |  |  |  |
| Overall CVD | 0.88 | (0.84, 0.92) | p<0.001 | 0.78 | (0.73, 0.82) | p<0.001 |
| Myocardial infarction | 0.76 | (0.70, 0.83) | p<0.001 | 0.67 | (0.60, 0.74) | p<0.001 |
| Heart failure | 0.88 | (0.82, 0.95) | 0.001 | 0.85 | (0.77, 0.93) | p<0.001 |
| Stroke | 0.91 | (0.85, 0.97) | 0.003 | 0.79 | (0.73, 0.86) | p<0.001 |
| Death | 0.84 | (0.80, 0.88) | p<0.001 | 0.63 | (0.59, 0.67) | p<0.001 |
| Muscle related AE | 0.90 | (0.78, 1.05) | 0.184 | 0.80 | (0.66, 0.96) | 0.016 |
| Liver dysfunction | 0.96 | (0.90, 1.03) | 0.284 | 0.87 | (0.80, 0.95) | 0.002 |
| **Aged ≥85 years** |  |  |  |  |  |  |
| Overall CVD | 0.78 | (0.69, 0.88) | p<0.001 | 0.70 | (0.61, 0.80) | p<0.001 |
| Myocardial infarction | 0.88 | (0.70, 1.10) | 0.253 | 0.84 | (0.65, 1.09) | 0.181 |
| Heart failure | 0.70 | (0.58, 0.85) | p<0.001 | 0.66 | (0.54, 0.82) | p<0.001 |
| Stroke | 0.70 | (0.59, 0.83) | p<0.001 | 0.64 | (0.53, 0.77) | p<0.001 |
| Death | 0.77 | (0.69, 0.86) | p<0.001 | 0.62 | (0.55, 0.71) | p<0.001 |
| Muscle related AE | 1.05 | (0.66, 1.67) | 0.843 | 1.17 | (0.68, 2.03) | 0.565 |
| Liver dysfunction | 0.92 | (0.76, 1.12) | 0.425 | 0.78 | (0.62, 0.97) | 0.028 |

Analyses adjusted for sex, age, smoking status, hemoglobin A1c, low-density lipoprotein cholesterol, high-density lipoprotein cholesterol, total cholesterol, estimated glomerular filtration rate, comorbidities (including Charlson Comorbidity Index, hypertension, peripheral vascular disease, chronic obstructive pulmonary disease, atrial fibrillation, renal disease, dementia, obesity), drug use (aspirin, insulin, oral antidiabetic drugs, angiotensin-converting enzyme (ACE) inhibitors, β-blockers, calcium channel blockers, diuretics), baseline calendar month, month of follow-up and its square term, Specialist Out-Patient Clinic attendance (within 1 year before baseline) and hospitalization (within 1 year before baseline).

**Table Q. Sensitivity analysis of the primary composite outcome of major cardiovascular diseases without heart failure**

|  | **ITT analysis** | | **Per-protocol analysis** | |
| --- | --- | --- | --- | --- |
|  | **Hazard ratio**  **(95%CI)** | **p-value** | **Hazard ratio**  **(95%CI)** | **p-value** |
| **Age 60-74 years** | 0.85 (0.82, 0.89) | p<0.001 | 0.72 (0.67, 0.77) | p<0.001 |
| **Age 75-84 years** | 0.87 (0.82, 0.91) | p<0.001 | 0.72 (0.66, 0.78) | p<0.001 |
| **Age ≥ 85 years** | 0.85 (0.74, 0.98) | 0.023 | 0.64 (0.52, 0.78) | p<0.001 |

Analyses adjusted for sex, age, smoking status, hemoglobin A1c, low-density lipoprotein cholesterol, high-density lipoprotein cholesterol, total cholesterol, estimated glomerular filtration rate, comorbidities (including Charlson Comorbidity Index, hypertension, peripheral vascular disease, chronic obstructive pulmonary disease, atrial fibrillation, renal disease, dementia, obesity), drug use (aspirin, insulin, oral antidiabetic drugs, angiotensin-converting enzyme (ACE) inhibitors, β-blockers, calcium channel blockers, diuretics), baseline calendar month, month of follow-up and its square term, Specialist Out-Patient Clinic attendance (within 1 year before baseline) and hospitalization (within 1 year before baseline).

**Table R. Sensitivity analysis of excluding the diagnosis code of non-incident events in case definition of outcome events**

|  | **ITT analysis** | | | | **Per-protocol analysis** | | | |
| --- | --- | --- | --- | --- | --- | --- | --- | --- |
|  | **Number of events** | | **Hazard ratios** | **p-value** | **Number of events** | | **Hazard ratios** | **p-value** |
|  | Initiators | Non-initiators |  |  | Initiators | Non-initiators |  |  |
| **Age 60-74 years** |  |  |  |  |  |  |  |  |
| MI (without ICD-9 code 412) | 1364 | 1591 | 0.83 (0.77, 0.89) | p<0.001 | 1051 | 819 | 0.70 (0.62, 0.79) | p<0.001 |
| Stroke (without ICD-9 code 438.x) | 2942 | 3281 | 0.87 (0.83, 0.91) | p<0.001 | 2349 | 1799 | 0.75 (0.69, 0.82) | p<0.001 |
| **Age 75-84 years** |  |  |  |  |  |  |  |  |
| MI (without ICD-9 code 412) | 915 | 1100 | 0.78 (0.71, 0.85) | p<0.001 | 724 | 716 | 0.63 (0.55, 0.72) | p<0.001 |
| Stroke (without ICD-9 code 438.x) | 1723 | 1826 | 0.90 (0.85, 0.97) | 0.003 | 1347 | 1161 | 0.75 (0.68, 0.84) | p<0.001 |
| **Age ≥ 85 years** |  |  |  |  |  |  |  |  |
| MI (without ICD-9 code 412) | 160 | 169 | 0.86 (0.69, 1.09) | 0.215 | 129 | 127 | 0.72 (0.52, 0.99) | 0.045 |
| Stroke (without ICD-9 code 438.x) | 235 | 255 | 0.83 (0.69, 1.00) | 0.045 | 197 | 217 | 0.65 (0.50, 0.84) | 0.001 |

Analyses adjusted for sex, age, smoking status, hemoglobin A1c, low-density lipoprotein cholesterol, high-density lipoprotein cholesterol, total cholesterol, estimated glomerular filtration rate, comorbidities (including Charlson Comorbidity Index, hypertension, peripheral vascular disease, chronic obstructive pulmonary disease, atrial fibrillation, renal disease, dementia, obesity), drug use (aspirin, insulin, oral antidiabetic drugs, angiotensin-converting enzyme (ACE) inhibitors, β-blockers, calcium channel blockers, diuretics), baseline calendar month, month of follow-up and its square term, Specialist Out-Patient Clinic attendance (within 1 year before baseline) and hospitalization (within 1 year before baseline).

**Figure A. Distribution of the propensity score**

Aged 60-74 years

**
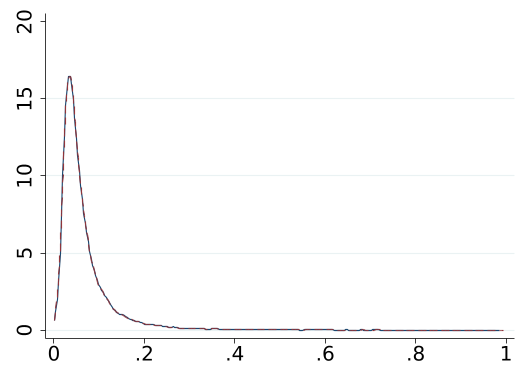
**

**
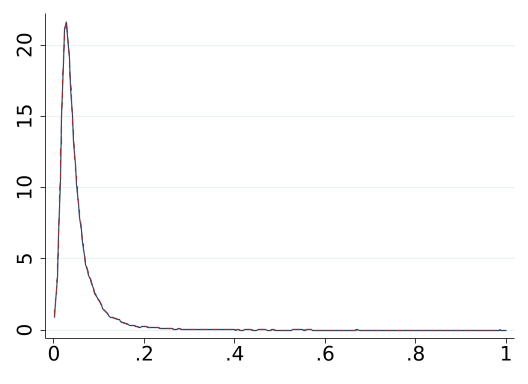
**

Aged 75-84 years

**
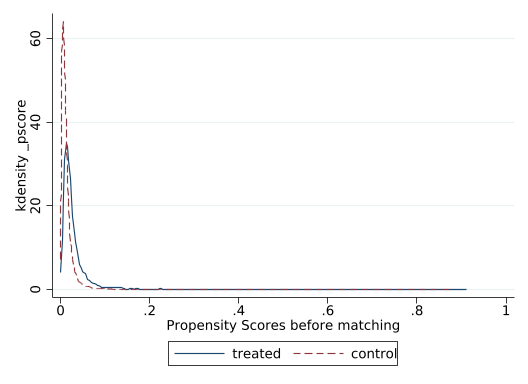

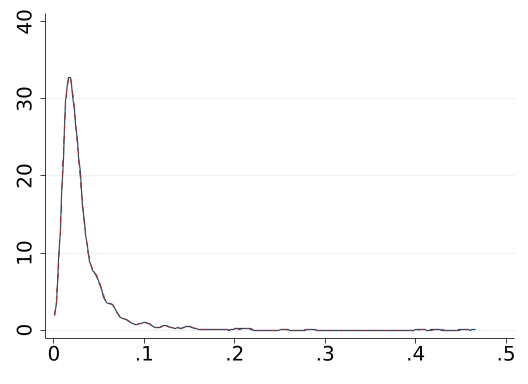
**

Aged ≥ 85 years

**Figure B. Distribution of weight distribution by treatment strategies**

**
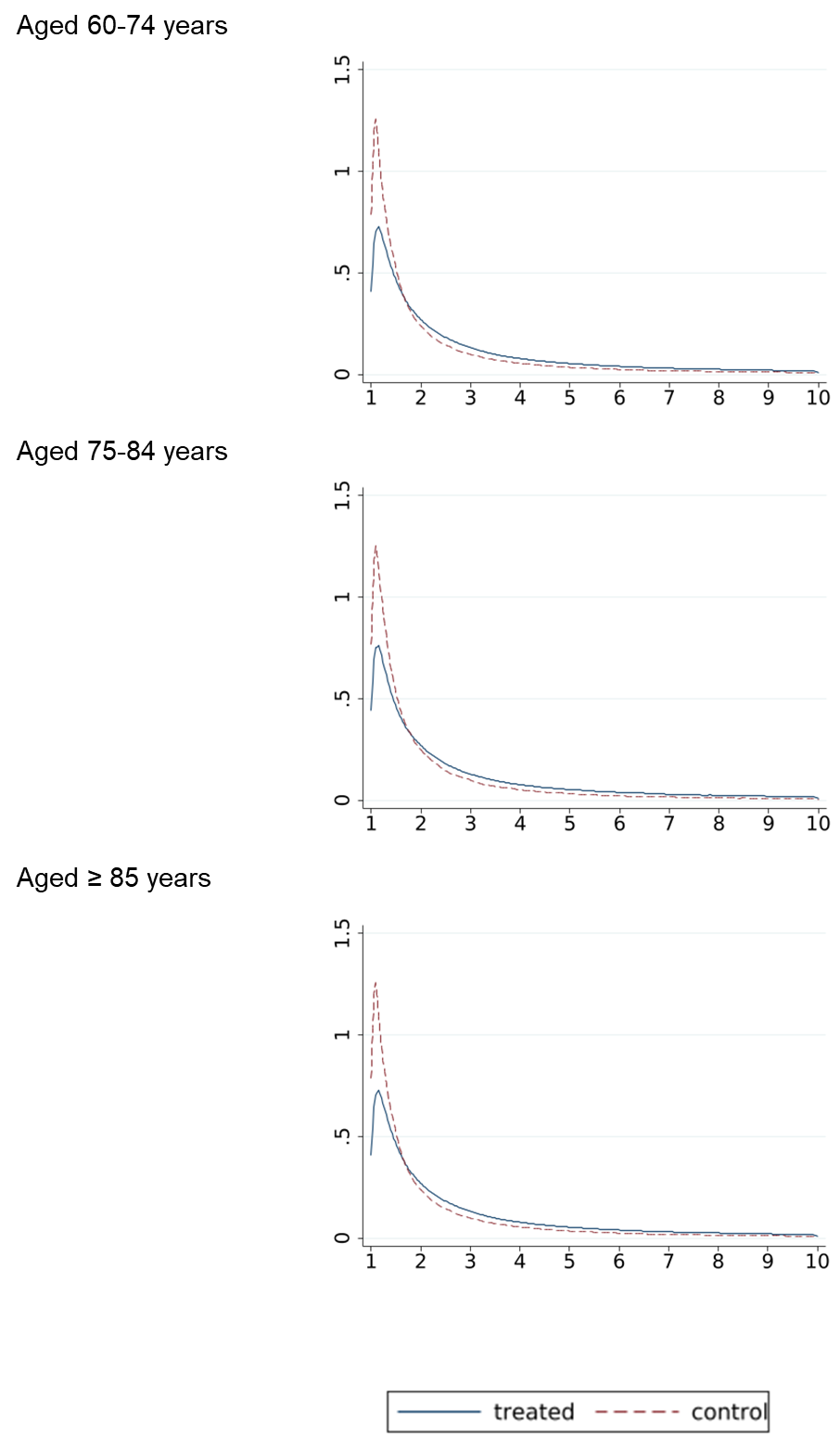
**
